# Gradient-Robust Hybrid DG Discretizations for the Compressible Stokes Equations

**DOI:** 10.1007/s10915-024-02605-2

**Published:** 2024-07-04

**Authors:** P. L. Lederer, C. Merdon

**Affiliations:** 1 Department of Applied Mathematics, University of Twente, Hallenweg 19, 7522NH Enschede, Netherlands; 2 Weierstrass Institute for Applied Analysis and Stochastics, Mohrenstr. 39, 10117 Berlin, Germany

**Keywords:** Compressible Stokes equations, Hybrid discontinuous Galerkin methods, Well-balanced schemes, Gradient-robustness

## Abstract

This paper studies two hybrid discontinuous Galerkin (HDG) discretizations for the velocity-density formulation of the compressible Stokes equations with respect to several desired structural properties, namely provable convergence, the preservation of non-negativity and mass constraints for the density, and gradient-robustness. The later property dramatically enhances the accuracy in well-balanced situations, such as the hydrostatic balance where the pressure gradient balances the gravity force. One of the studied schemes employs an $H(\textrm{div})$-conforming velocity ansatz space which ensures all mentioned properties, while a fully discontinuous method is shown to satisfy all properties but the gradient-robustness. Also higher-order schemes for both variants are presented and compared in three numerical benchmark problems. The final example shows the importance also for non-hydrostatic well-balanced states for the compressible Navier–Stokes equations.

## Introduction

For incompressible flows the concept of pressure-robustness characterizes discretizations that allow for a priori velocity error estimates that are independent of the pressure and the viscosity parameter. Otherwise the scheme can suffer from a severe locking phenomenon [15, 21, 31, 38]. A lack of pressure-robustness can be avoided by using divergence-free schemes, e.g., [9, 12, 18–20, 25, 37, 39], or, alternatively, non pressure-robust classical discretizations can be ’repaired’ by applying $H(\textrm{div})$-conforming reconstruction operators at critical spots [24, 31–33].

Here, we consider the non-conservative form of the compressible Stokes model problem that seeks a velocity $\varvec{u}$ (with homogeneous Dirichlet boundary data for simplicity) and a non-negative density $\varrho$ with a mass constraint such that

1 $$\begin{aligned} -\nu \varDelta \varvec{u} + \nabla p(\varrho )&= \varrho \varvec{g} + \varvec{f}, \end{aligned}$$

2 $$\begin{aligned} \textrm{div}(\varrho \varvec{u})&= 0, \end{aligned}$$

for a given equation of state, e.g. the ideal gas law $p(\varrho ) = {c_M}\varrho$. Here, $\nu$ is the viscosity and $c_M$ is a constant related to the inverse of the squared Mach number and the (assumed constant) temperature. In [1] the authors, inspired by [14] and [13], extended the concept of pressure-robustness to the compressible Stokes equations and connected it with the concept of well-balanced schemes. As in the incompressible case, dominant gradient fields in the momentum balance can appear, and methods that do not suffer from this are coined gradient-robust (since the gradient force could be also balanced by $\nabla (\textrm{div}(\varvec{u}))$ and not only by the pressure). In the compressible setting, well-balanced states beyond $\nabla p(\varrho ) = \nabla \varvec{f}$ can appear, in particular $\nabla p(\varrho ) = \varrho \varvec{g}$ in presence of the gravity term or other conservative forces. More well-balanced and non-hydrostatic states are possible, e.g., when including the convection term $\textrm{div}(\varrho \varvec{u} \otimes \varvec{u})$ or the geostrophic balance in presence of the Coriolis force $2 \varrho (\varOmega \times \varvec{u})$. It is non-trivial for numerical schemes to preserve these states accurately.

There are several approaches in the literature to design well-balanced schemes, mostly in the context of hyperbolic conservation laws and model problems like the shallow water equations with bottom topography or the Euler equations with gravity, see e.g. [2, 16, 17, 35, 36] and references therein. A popular approach in these references is a certain modification of the source term based on a hydrostatic reconstruction, i.e. a transformation to a set of variables that stays constant in the well-balanced state. An equivalent strategy from [3] requires a sufficiently accurate representation of the well-balanced state and then computes the deviations from this state.

In [1] the gradient-robustness property of a scheme was identified as one important ingredient for well-balancedness on general meshes. To do so, an inf-sup stable Bernardi–Raugel finite element method was coupled with a finite-volume method for the continuity equation. Moreover, a reconstruction operator that preserves the discrete divergence of the test function was employed in the gravity term, ensuring that the discretely divergence-free part of the solution is really divergence-free and therefore orthogonal onto gradient forces. This was the key ingredient to ensure gradient-robustness and therefore a certain well-balancedness. The scheme also ensures the non-negativity constraint for the density and guaranteed convergence and is asymptotic-preserving in the sense that it converges to a pressure-robust scheme for the incompressible Stokes equations if the Mach number goes to zero or, equivalently, if ${c_M}$ goes to infinity. In [34] an unconditional error estimate for the pressure of that scheme for the semi-stationary compressible Stokes problem and a similar discrete scheme was shown. However, here some additional stabilization terms in the continuity equation were added which unfortunately compromise the gradient-robustness.

As for pressure-robust and divergence-free methods for incompressible flows, the concept of gradient-robustness is based on discrete exact sequences or De Rham complexes, which ensure the structure-preserving features of the method. Another identical concept is the framework of compatible ($H(\textrm{div})$-conforming) FEM, see e.g. [10] where it is applied to the Euler equations and shallow water equations. In the present paper, hybrid discontinuous Galerkin schemes are explored that avoid the introduction of a reconstruction operator as in [1] and straightforwardly allow for higher order schemes. Note, that an extension of the model problem (1) to a model with the full elasticity tensor $-\nu \textrm{div} (\mathbb {C} \varepsilon (\varvec{u}))$ is straightforward and requires an additional Korn inequality to hold. For the discontinuous Galerkin methods discussed here the necessary estimates can be found in [5], or in the context of mixed FEM for linear elasticity [28].

Two variants of the hybrid discontinuous Galerkin (HDG) methods are studied and their lowest order versions are shown to converge and preserve non-negativity and mass constraints on general meshes. Although the main line of arguments is similar to [1, 14], adaptations to the DG context are needed. Moreover a sharper stability estimate with respect to the gravity force $\varvec{g}$ is provided.

The first variant discretizes the velocity field in an $H(\textrm{div})$-conforming Brezzi–Douglas–Marini (BDM) space, which allows that the discretely divergence-free part of the velocity is exactly divergence-free. Therefore, it is perfectly orthogonal on any gradient in the momentum balance. Thus, no $H(\textrm{div})$-conforming interpolation as in [1] is needed, but requires a different discretization of the diffusive term instead. In the following let $q, \psi$ be smooth scalar fields. By $L^2$-orthogonality of divergence-free velocity fields and gradients, $\varvec{f} = \nabla q$ yields a well-balanced discrete solution with $\varvec{u}=\varvec{0}$. Also a gravity-related balanced state with $\varrho \varvec{g} = \varrho \nabla \psi = \nabla q$ is approximated much better than without gradient-robustness. However, the discretization of $\varrho$ by $\varrho _h$ generates a small perturbation $(\varrho - \varrho _h) \varvec{g}$ that may not be fully irrotational and therefore may cause an imbalance and spurious oscillations that scale with $1/(c_M\nu )$. This was also observed in [1]. The second variant of the HDG method also relaxes the $H(\textrm{div})$-conformity and therefore the divergence-constraint of the velocity is formulated in the spirit of the DG versions from [11]. While this also allows for a provably converging and non-negativity-preserving scheme, the relaxation of the divergence-constraint compromises the gradient-robustness and, in consequence, also the well-balancedness. Numerical examples confirm in which situations the $H(\textrm{div})$-conforming scheme is superior, namely for low Mach numbers and small $\nu$. Moreover, the last example demonstrates the importance of gradient-robustness for non-hydrostatic well-balanced states like $\varrho \textrm{div}(\varvec{u} \otimes \varvec{u}) + \nabla p(\varrho ) = \varvec{0}$ in the compressible Navier–Stokes setting, where the convection term can be a gradient.

The rest of the paper is structured as follows. Section 2 introduces the model problem and basic notation and concepts. Section 3 introduces the gradient-robust HDG scheme. Section 4 proves stability and existence of discrete solutions. Section 5 shows convergence of the gradient-robust scheme. Section 6 shortly discusses the fully discontinuous variant and the necessary modifications to the stability and convergence proof. Section 7 compares both variants in three numerical examples with a focus on the benefits of gradient-robustness.

## Preliminaries

In the following and for the rest of this work we consider a Lipschitz domain $\varOmega \subset \mathbb {R}^d$ with $d = 2$ or $d=3$. For a subset $\omega \subseteq \varOmega$ we use $( \bullet , \bullet )_\omega$ to denote the $L^2$ inner product on $\omega$, with $\Vert \bullet \Vert ^2_\omega = ( \bullet , \bullet )_\omega$. For $\omega = \varOmega$ we omit the subscript, i.e. use $( \bullet , \bullet )_\varOmega =( \bullet , \bullet )$ and $\Vert \bullet \Vert _\varOmega = \Vert \bullet \Vert$. We employ standard notation of Sobolev spaces and use bold symbols for their vector valued versions, e.g. $H^1(\varOmega )$ and $\varvec{H}^1(\varOmega ) = [H^1(\varOmega )]^d$ for the first order Sobolev spaces in one and *d* dimensions, respectively. Moreover, we use the common notation $H(\textrm{div}, \varOmega )$ (i.e. without a bold symbol) to denote the (vector-valued) Sobolev space of functions whose weak-divergence is in $L^2$. Finally note that we make use of a zero index to denote a vanishing trace on $\partial \varOmega$ of the corresponding (continuous) trace operator.

### The Compressible Stokes Model Problem

Let $\varvec{g} \in \varvec{L}^\infty (\varOmega )$ be a given gravity force, and additionally, for conceptual purposes, consider a second force $\varvec{f} \in \varvec{L}^2(\varOmega )$.

The weak formulation of the compressible Stokes equations seeks $\varvec{u} \in \varvec{V}:= \varvec{H}^1_0(\varOmega )$ and $\varrho \in Q:= L^2(\varOmega )$ such that

3a $$\begin{aligned} a(\varvec{u}, \varvec{v}) + b(\varrho , \varvec{v})&= (\varrho \varvec{v}, \varvec{g}) + (\varvec{v}, \varvec{f}){} & {} \text {for all } \varvec{v} \in \varvec{V}, \end{aligned}$$

3b $$\begin{aligned} c(\varrho , \varvec{u}, \lambda )&= 0{} & {} \text {for all } \lambda \in W^{1, \infty }(\varOmega ), \end{aligned}$$

where

$$\begin{aligned} a(\varvec{u},\varvec{v})&:= \nu (\nabla \varvec{u}, \nabla \varvec{v}),\\ b(\varrho , \varvec{v})&:= -(p(\varrho ), \textrm{div}(\varvec{v})),\\ c(\varrho , \varvec{v}, \lambda )&:= (\varrho \varvec{v}, \nabla \lambda ). \end{aligned}$$

Throughout the paper we assume a linear equation of state and a mass constraint given by

4 $$\begin{aligned} p(\varrho ) = {c_M}\varrho , \quad \text {and} \quad (\varrho ,1) = M, \end{aligned}$$

where $M > 0$ and ${c_M}$ are constants. The later can be considered as the squared inverse of the Mach number, i.e., ${c_M}\approx {M\!a}^{-2}$.

### Gradient Forces and Hydrostatic/Well-Balanced Solutions

This section is concerned with a proper characterization of gradient-robustness and well-balancedness. Both concepts are related to gradient fields in the momentum balance.

For the incompressible Stokes problem one observes that any gradient force $\varvec{f} = \nabla q$, with a given potential *q*, leads to a hydrostatic solution $\varvec{u} \equiv \varvec{0}$ and a pressure $\nabla p = \nabla q$ that fully balances $\varvec{f}$. A numerical method that preserves this was coined pressure-robust [21, 31]. The correct balancing of the gradient force $\nabla q$ exploits the $L^2$-orthogonality of divergence-free functions on $\nabla q$, i.e.,

$$\begin{aligned} (\nabla q, \varvec{v}) = - (q, \textrm{div} (\varvec{v})) = 0, \qquad \text {for all } \varvec{v} \in \varvec{V}_0 := \lbrace \varvec{v} \in \varvec{V} : \textrm{div} (\varvec{v} )= 0 \rbrace . \end{aligned}$$

In the present compressible setting given by (3) and assuming $\varvec{f} = 0$ a similar hydrostatic balance is possible, namely

5 $$\begin{aligned} \nabla p(\varrho ) = \varrho \varvec{g} = \varrho \nabla \psi . \end{aligned}$$

This situation appears, e.g., in an atmosphere-at-rest-scenario and might be considered equivalent to the lake-at-rest scenario in shallow water equations with bottom topography [35]. A discrete scheme that correctly balances gradient forces $\psi$ and computes hydrostatic solutions with $\varvec{u} = \varvec{0}$ in these cases is called well-balanced.

For the equation of state $p(\varrho ) = {c_M}\varrho$ the hydrostatic balance can be reformulated to

6 $$\begin{aligned} \nabla p(\varrho ) = {c_M}\varrho \nabla (\log \varrho ) = \varrho \varvec{g} = \varrho \nabla \psi . \end{aligned}$$

This yields (uniformly positive) solutions of the form $\varrho := \varrho _0 \exp (\psi /{c_M})$ where the constant $\varrho _0$ is chosen such that the mass constraint is satisfied.

It is non-trivial for a discrete scheme to compute hydrostatic solutions in this case without using a priori information. Indeed, one could subtract the exact solution from the equation and compute a deviation density, in the spirit of, e.g., [3]. However, in more complex situations, e.g. other forces, multi-physics or boundary conditions or different equations of state $p(\varrho )$, analytical solutions might be unavailable. Hence, here we are interested in an out-of-the-box scheme that is as accurate as possible without a priori modifications.

The purpose of the forcing $\varvec{f}$ in (3) is to better explain the importance of gradient-robustness as an important ingredient for well-balancedness. To this end let us consider a gradient force $\varvec{f} = \nabla q$ and $\varvec{g} = 0$ and the hydrostatic balance

7 $$\begin{aligned} \nabla p(\varrho ) = \varvec{f} = \nabla q. \end{aligned}$$

Due to the non-negativity and mass constraint, this balance (and therefore a hydrostatic solution) is only satisfied if one can choose a constant *C* such that the density is given by

$$\begin{aligned} \varrho := (q - C)/{c_M}, \end{aligned}$$

and at the same time stays non-negative and satisfies $((q - C)/{c_M}, 1) = M$. This is only possible if *q* is small enough or *M* is large enough and such forces $\varvec{f}$ are called admissible, see also [1, Lemma 4.3] for a motivation.

To summarize we consider these two qualities of well-balancedness for this model problem:

- a scheme for (3) is said to be gradient-robust if it admits a hydrostatic solution whenever $\varvec{g} = \varvec{0}$ and $\varvec{f}$ is an admissible gradient force;
- a scheme for (3) is said to be well-balanced if it admits a hydrostatic solution whenever $\varvec{f} = \varvec{0}$ and $\varvec{g}$ is a gradient force.

#### Remark 1

If $\varrho$ is the exact solution for given $\varvec{g} = \nabla \psi$ and $M > 0$, then $\varvec{f}:= \varrho \nabla \psi$ is always an admissible force. This follows by the calculation above. A comparison of the two force terms implies that the lack of well-balancedness of a gradient-robust scheme is therefore caused by or determined by the non-irrotational part of $(\varrho - \varrho _h) \nabla \psi$, where $\varrho _h$ is the density approximation of the scheme, whereas the irrotational part of that quantity is treated correctly by a gradient-robust scheme.

In [1] gradient-robustness was characterized by the observation, that the divergence-free part of the discrete velocity solution satisfies a pressure-robust discretization of an incompressible Stokes problem. Key to this is a correct balancing of divergence-free forces and gradient forces via structural properties of $H(\textrm{div})$-conforming finite element spaces, namely the $L^2$-orthogonality of divergence-free functions and gradients like the force terms discussed above.

## A Gradient-Robust HDG Scheme

This section discusses a hybrid discontinuous Galerkin (HDG) discretization for the compressible Stokes equation where the discrete velocity $\varvec{u}_h$ is $H(\textrm{div})$-conforming. This implies gradient-robustness and asymptotic convergence to a pressure-robust discretization of the incompressible Stokes problem when the Mach number tends to zero $M\!a \rightarrow 0$.

### Notation

Consider a regular triangulation $\mathcal {T}$ of $\varOmega$ into simplices. The set of vertices is given by $\mathcal {N}$ and the sets of faces by $\mathcal {F}$. For simplification we further assume that $\mathcal {T}$ is quasi uniform and use $h_T:= {\text {diam}}(T)$, $h_F:= {\text {diam}}(F)$ for $T \in \mathcal {T}$ and $F \in \mathcal {F}$ and define $h:=\max \limits _{T \in \mathcal {T}} h_T$. Due to quasi uniformity we have $h \approx h_T \approx h_F$.

On a face $F \in \mathcal {F}$ we define a unit vector normal $\varvec{n}_F$ with an arbitrary but fixed orientation. Note, that the (fixed) orientation of $\varvec{n}_F$ also defines the orientation of the jump operator $[\![{\cdot }]\!]$, e.g. let $F = T_1 \cap T_2$ for two elements $T_1$ and $T_2$ and fix $\varvec{n}_F$ to point from $T_1$ to $T_2$, then we have on *F*

$$\begin{aligned}{}[\![{q}]\!] := q_{h,1} - q_{h,2}, \quad \text {with} \quad q_{h,i} := q_h|_{T_i}, \text { for } i = 1,2. \end{aligned}$$

On the domain boundary and on boundaries $\partial T$ for $T \in \mathcal {T}$ we use $\varvec{n}$ to denote the outward pointing normal vector. Further, we define the tangential projection for a function $\varvec{v}$ by $\varvec{v}_t:= \varvec{v} - (\varvec{v} \cdot \varvec{n}) \varvec{n}$. Further, on the domain boundary we have $\varvec{n}$ = $\varvec{n}_F$, and the jump operator equals the identity. We denote by $P^k(\omega )$ the set of polynomials on $\omega \subset \varOmega$ of total order *k*, and again use bold symbols to denote the corresponding vector-valued versions.

### The $H(\textrm{div})$-HDG Scheme

Consider the finite element spaces

$$\begin{aligned} \varvec{V}_h&:= \varvec{P}_{k}(\mathcal {T}) \cap H_0(\textrm{div}, \varOmega ),\\ \widehat{\varvec{V}}_h&:= \left\{ \widehat{\varvec{v}}_h \in \varvec{L}^2(\mathcal {F}): \widehat{\varvec{v}}_h|_F \in \varvec{P}_{k}(F), \widehat{\varvec{v}}_h \cdot \varvec{n}_F = 0 ~\forall F \in \mathcal {F}, \widehat{\varvec{v}}_h = 0 \text { on } \partial \varOmega \right\} ,\\ Q_h&:= P_{k-1}(\mathcal {T}). \end{aligned}$$

Here, $\varvec{V}_h$ is the $H(\textrm{div})$-conforming BDM space of order *k* which is used as velocity ansatz space, and $Q_h$ is the discontinuous ansatz space for the pressure and density. The hybridization space $\widehat{\varvec{V}}_h$ is used to couple the discontinuous tangential parts of discrete velocities in $\varvec{V}_h$ in a hybrid DG fashion. Thus, $\widehat{\varvec{V}}_h$ can be seen as as the ansatz space for the tangential traces of velocities on the skeleton $\mathcal {F}$.

The suggested discrete scheme seeks $\left( (\varvec{u}_h, \widehat{\varvec{u}}_h), \varrho _h\right) \in (\varvec{V}_h \times \widehat{\varvec{V}}_h) \times Q_h$ such that

8a $$\begin{aligned} a_h((\varvec{u}_h, \widehat{\varvec{u}}_h),(\varvec{v}_h, \widehat{\varvec{v}}_h)) + b(p(\varrho _h), \varvec{v}_h)&= F_h(\varvec{v}_h) + G_h(\varrho _h, \varvec{v}_h), \end{aligned}$$

8b $$\begin{aligned} c_h(\varrho _h, \varvec{u}_h, \lambda _h)&= 0, \end{aligned}$$

8c $$\begin{aligned} (\varrho _h,1)&= M, \end{aligned}$$

for all $(\varvec{v}_h, \widehat{\varvec{v}}_h) \in \varvec{V}_h \times \widehat{\varvec{V}}_h$ and $\lambda _h \in Q_h$. Here, the forms are defined by

$$\begin{aligned} a_h((\varvec{u}_h, \widehat{\varvec{u}}_h),(\varvec{v}_h, \widehat{\varvec{v}}_h))&:= \nu \sum _{T \in \mathcal {T}} (\nabla \varvec{u}_h, \nabla \varvec{v}_h)_T +(\nabla \varvec{u}_h \varvec{n}, (\widehat{\varvec{v}}_h - \varvec{v}_h)_t)_{\partial T}\\&\quad + (\nabla \varvec{v}_h \varvec{n}, (\widehat{\varvec{u}}_h - \varvec{u}_h)_t)_{\partial T} + \frac{\alpha k^2}{h} ((\widehat{\varvec{u}}_h - \varvec{u}_h)_t, (\widehat{\varvec{v}}_h - \varvec{v}_h)_t)_{\partial T},\\ c_h(\varrho _h, \varvec{u}_h, \lambda _h)&:=-\sum _{T \in \mathcal {T}} (\varrho _h \varvec{u}_h, \nabla \lambda _h)_T + (\varvec{u}_h\cdot \varvec{n} \varrho _h^{up}, \lambda _h)_{\partial T}, \\ G_h(\varrho _h, \varvec{v}_h)&:= (\varvec{g}, \varrho _h \varvec{v}_h),\\ F_h(\varvec{v}_h)&:= (\varvec{f}, \varvec{v}_h), \end{aligned}$$

where $\varrho _h^{up}$ is the standard upwind value associated to $\varvec{u}_h$. The vanishing Dirichlet value of the velocity was incorporated in an essential (direct) manner for the normal part via $\varvec{V}_h$, and in a weak (DG-like) manner for the tangential part via $\widehat{\varvec{V}}_h$. As usual for (hybrid) DG methods, the parameter $\alpha > 0$ has to be chosen sufficiently large enough. In our numerical examples we always choose $\alpha = 10$. For a comparison and discussion on DG and HDG methods we refer to the literature, e.g. [7, 8, 30]. Note that an alternative formulation of the diffusive fluxes would be possible by means of a mixed formulation which is inherently stable (without choosing an $\alpha >0$). See [23] for more details and a comparison with respect to the stabilization parameter. For the sake of simplicity we do not use this formulation in this work. The proposed scheme of this work is similar to the one in [1], but instead of an $H^1$-conforming Bernardi–Raugel method a hybrid $H(\textrm{div})$-DG approach is employed. A stability and convergence proof is given in Sects. 4 and 5.

The analysis involves the usual HDG norm

9 $$\begin{aligned} \Vert (\varvec{u}_h, {\widehat{\varvec{u}}}_h) \Vert ^2_{1,h}&:= \sum \limits _{T \in \mathcal {T}} \Vert \nabla \varvec{u}_h \Vert _T^2 + \frac{1}{h_T} \Vert (\varvec{u}_h - {\widehat{\varvec{u}}}_h)_t \Vert _{\partial T}^2. \end{aligned}$$

Moreover, we recall that the chosen velocity and pressure spaces allow for an LBB-condition with respect to the space

$$\begin{aligned} Q_h \cap L^2_0(\varOmega ) := \Bigl \{\lambda \in L^2(\varOmega ): \int _\varOmega \lambda = 0 \Bigr \}, \end{aligned}$$

which can be found for example in [27]. This is one important ingredient for the stability analysis of the scheme.

#### Lemma 1

(LBB-stability Stokes) There exists some $\beta > 0$ independent of *h*, such that for all $\lambda _h \in Q_h \cap L^2_0(\varOmega )$, we have

$$\begin{aligned} \sup _{\begin{array}{c} (\varvec{v}_h, {\widehat{\varvec{v}}}_h) \in {\varvec{V}}_h \times {\widehat{\varvec{V}}}_h\\ (\varvec{v}_h, {\widehat{\varvec{v}}}_h) \ne (\varvec{0}, \varvec{0}) \end{array}} \frac{b(\lambda _h, \varvec{v}_h)}{\Vert (\varvec{v}_h, {\widehat{ \varvec{v}}}_h) \Vert _{1,h} } \geqslant \beta \Vert \lambda _h \Vert . \end{aligned}$$

#### Lemma 2

(Gradient-robustness) The scheme (8) is gradient-robust.

#### Proof

Consider an admissable gradient force $\varvec{f} = \nabla q$ (in the sense of Sect. 2.2) and the incompressible Stokes problem: seek $(\varvec{u}^0_h, \widehat{\varvec{u}}^0_h)$ with $\textrm{div} (\varvec{u}^0_h) = 0$ and (up to an arbitrary global constant *C*) some $p_h \in Q_h$ such that

10 $$\begin{aligned} a_h((\varvec{u}^0_h, \widehat{\varvec{u}}^0_h),(\varvec{v}_h, \widehat{\varvec{v}}_h)) - (p_h, \textrm{div} (\varvec{v}_h))&= (\nabla q, \varvec{v}_h) = -(q, \textrm{div} (\varvec{v}_h)). \end{aligned}$$

Testing with $(\varvec{v}_h, \widehat{\varvec{v}}_h) = (\varvec{u}^0_h, \widehat{\varvec{u}}^0_h)$ yields

$$\begin{aligned} \Vert (\varvec{u}^0_h, \widehat{\varvec{u}}^0_h) \Vert ^2_{1,h} = 0. \end{aligned}$$

Standard Stokes pressure estimates show $p_h:= \varPi _{Q_h} q + C$, where $\varPi _{Q_h}$ is the $L^2$-projection onto $Q_h$. Due to the admissibility assumption of $\varvec{f}$ we can then find a constant *C* such that $\varrho _h:= p_h + C > 0$ also satisfies the mass constraint. Hence, $(\varvec{u}_h, \widehat{\varvec{u}}_h) = (\varvec{u}^0_h, \widehat{\varvec{u}}^0_h) = (\varvec{0},\varvec{0})$ and $\varrho _h$ also solves (8), thus the scheme admits a hydrostatic solution. $\square$

## Stability and Existence of Solutions

This section shows stability and existence of solutions as well as positivity and mass preservation of $\varrho _h$ for the suggested HDG scheme. However, only the lowest order case $k = 1$ guarantees the positivity preservation of $\varrho _h$.

### Stability

The following Lemma recalls [1, Lemma 4.2].

#### Lemma 3

Let $\varvec{u} \in L^2(\mathcal {F})$ be a single-valued function on each face of the triangulation, and let $\varrho ^{up}_h$ be the $\varvec{u}$-associated upwind value. For any twice continuously differentiable convex function $\phi : [0, \infty ) \rightarrow \mathbb {R}^{+}$ there holds

11 $$\begin{aligned}{} & {} \sum _{T \in \mathcal {T}} \int _{\partial T} \varvec{u} \cdot \varvec{n} \varrho _h^{up} \phi ^\prime (\varrho _h) - \sum _{T \in \mathcal {T}} \int _{\partial T} \varvec{u} \cdot \varvec{n} (\varrho _h \phi ^\prime (\varrho _h) - \phi (\varrho _h)) \nonumber \\{} & {} \quad =\frac{1}{2} \sum _{F \in \mathcal {F}} \phi ^{\prime \prime }(\varrho ^{F}_h) \Big | \int _F \varvec{u} \cdot \varvec{n}_F [\![{\varrho _h}]\!]^2 \Big | \geqslant 0, \end{aligned}$$

with intermediate values $\varrho _h^{F} \in [\min (\varrho _{h,1},\varrho _{h,2}),\max (\varrho _{h,1},\varrho _{h,2})]$ where $\varrho _{h,1}$ and $\varrho _{h,2}$ are the restrictions of $\varrho$ on the adjacent elements of facet *F*.

#### Proof

First note that the left difference can be written as

$$\begin{aligned}{} & {} \sum _{T \in \mathcal {T}} \int _{\partial T} \varvec{u} \cdot \varvec{n} \varrho _h^{up} \phi ^\prime (\varrho _h) - \sum _{T \in \mathcal {T}} \int _{\partial T} \varvec{u} \cdot \varvec{n} (\varrho _h \phi ^\prime (\varrho _h) - \phi (\varrho _h)) \\{} & {} \quad = \sum _{T \in \mathcal {T}} \int _{\partial T} \varvec{u} \cdot \varvec{n} (\varrho _h^{up} \phi ^\prime (\varrho _h) - \varrho _h \phi ^\prime (\varrho _h) + \phi (\varrho _h)). \end{aligned}$$

Let $T_1$ and $T_2$ be the two neighboring elements of a facet $F \in \mathcal {F}$, then we can rewrite the sum above as

$$\begin{aligned}{} & {} \sum _{T \in \mathcal {T}} \int _{\partial T} \varvec{u} \cdot \varvec{n} (\varrho _h^{up} \phi ^\prime (\varrho _h) - \varrho _h \phi ^\prime (\varrho _h) + \phi (\varrho _h)) \\{} & {} \quad = \sum _{F \in \mathcal {F}} \int _{F} \varvec{u} \cdot \varvec{n}_F (\phi ^\prime (\varrho _{h,1}) (\varrho _h^{up} - \varrho _{h,1}) + \phi ^\prime (\varrho _{h,2}) (\varrho _{h,2} - \varrho _h^{up}) + \phi (\varrho _{h,1}) - \phi (\varrho _{h,2})). \end{aligned}$$

Let $\theta _F = (\phi ^\prime (\varrho _{h,1}) (\varrho _h^{up} - \varrho _{h,1}) + \phi ^\prime (\varrho _{h,2}) (\varrho _{h,2} - \varrho _h^{up}) + \phi (\varrho _{h,1}) - \phi (\varrho _{h,2}))$. Consider the case $\int _F\varvec{u} \cdot \varvec{n}_F \geqslant 0$, then $\varrho _h^{up} - \varrho _{h,1}$, and thus with the Taylor expansion at $\varrho _{h,2}$, i.e.

$$\begin{aligned} \phi (\varrho _{h,1}) = \phi (\varrho _{h,2}) + \phi ^\prime (\varrho _{h,2}) (\phi (\varrho _{h,1}) - \phi (\varrho _{h,2})) + \frac{1}{2}\phi ^{\prime \prime }(\varrho _h^F) (\phi (\varrho _{h,1}) - \phi (\varrho _{h,2}))^2, \end{aligned}$$

with a point $\varrho _h^F \in [\min (\varrho _{h,1},\varrho _{h,2}),\max (\varrho _{h,1},\varrho _{h,2})]$, we get

$$\begin{aligned} \theta _F = \frac{1}{2}\phi ^{\prime \prime }(\varrho _h^F) (\phi (\varrho _{h,1}) - \phi (\varrho _{h,2}))^2 \geqslant 0, \end{aligned}$$

where the non-negativity follows by the convexity of $\phi$. For the other case, i.e. $\int _F\varvec{u} \cdot \varvec{n}_F < 0$, we derive similarly

$$\begin{aligned} \theta _F = -\frac{1}{2}\phi ^{\prime \prime }(\varrho _h^F) (\phi (\varrho _{h,1}) - \phi (\varrho _{h,2}))^2 \leqslant 0, \end{aligned}$$

and thus we get $\sum _{F \in \mathcal {F}} \int _{F} \varvec{u} \cdot \varvec{n}_F \theta _F \geqslant 0.$ $\square$

#### Theorem 1

(Stability) For the solution of (8), it holds

12 $$\begin{aligned} \Vert (\varvec{u}_h, {\widehat{ \varvec{u}}}_h) \Vert _{1,h}&\lesssim \nu ^{-1} \left( \Vert \varvec{f} \Vert + \Vert \varvec{g} \Vert _{L^\infty (\varOmega )} \Vert \varrho _h \Vert \right) , \end{aligned}$$

13 $$\begin{aligned} \sum _{F \in \mathcal {F}} \phi ^{\prime \prime }(\varrho ^{F}_h) \Big | \int _F \varvec{u}_h \cdot \varvec{n}_F [\![{\varrho _h}]\!]^2 \Big |&\lesssim \nu ^{-1} \left( \Vert \varvec{f} \Vert + \Vert \varvec{g} \Vert _{L^\infty (\varOmega )} \Vert \varrho _h \Vert \right) ^2,\end{aligned}$$

14 $$\begin{aligned} \Big (1 - \frac{C}{ {c_M}^2} \Vert \varvec{g} \Vert _{L^\infty (\varOmega )}^2\Big ) \Vert \varrho _h \Vert ^2&\lesssim \frac{1}{ {c_M}^2} \Vert \varvec{f} \Vert ^2 + M^2. \end{aligned}$$

Stability for $\varrho _h$ and $\varvec{u}_h$ is therefore guaranteed for $\Vert \varvec{g} \Vert _{L^\infty (\varOmega )} / {c_M}$ small enough. The constant *C* is a generic constant that depends on the shape of the cells, $|\varOmega |$ and $\alpha$, but not on *h*, ${c_M}$, *M* or $\nu$.

#### Proof

Following [9, 30] we have the coercivity estimate

15 $$\begin{aligned} \nu \Vert (\varvec{u}_h, {\widehat{ \varvec{u}}}_h) \Vert ^2_{1,h} \lesssim a((\varvec{u}_h, {\widehat{ \varvec{u}}}_h),(\varvec{u}_h, {\widehat{ \varvec{u}}}_h)). \end{aligned}$$

Testing the momentum equation with $(\varvec{v}_h, {\widehat{ \varvec{v}}}_h) = (\varvec{u}_h, {\widehat{ \varvec{u}}}_h)$ gives

$$\begin{aligned} a((\varvec{u}_h, {\widehat{ \varvec{u}}}_h),(\varvec{u}_h, {\widehat{ \varvec{u}}}_h)) + b(p(\varrho _h), \varvec{u}_h) = F_h(\varvec{u}_h) + G_h(\varrho _h,\varvec{u}_h). \end{aligned}$$

Due to the upwinding we get the correct sign from Lemma 3. For this choose the convex function $\phi (s) = {c_M}s \log (s)$, with $\phi ^\prime (s) = {c_M}(\log (s) + 1)$, then we have $\varrho _h \phi ^\prime (\varrho _h) - \phi (\varrho _h) = {c_M}\varrho _h = p(\varrho _h)$. By that (11) reads as

$$\begin{aligned} c_h(\varrho _h, \varvec{u}_h, -{c_M}(1 + \log (\varrho _h))) + b(p(\varrho _h), \varvec{u}_h) = \frac{1}{2} \sum _{F \in \mathcal {F}} \phi ^{\prime \prime }(\varrho ^{F}_h) \Big | \int _F \varvec{u}_h \cdot \varvec{n}_F [\![{\varrho _h}]\!]^2 \Big |. \end{aligned}$$

With (8b) and ${c_M}(1 + \log (\varrho _h)) \in Q_h$, we get $b(p(\varrho _h), \varvec{u}_h) \geqslant 0$. It remains to bound the right-hand side.

Using a discrete Friedrichs-type inequality, see for example [6], we get

$$\begin{aligned} F_h(\varvec{u}_h) \lesssim \Vert \varvec{f} \Vert \Vert (\varvec{u}_h, {\widehat{ \varvec{u}}}_h) \Vert _{1,h}. \end{aligned}$$

For the other right-hand side term we get similarly

$$\begin{aligned} G_h(\varrho _h,\varvec{u}_h) = (\varvec{g}, \varrho _h \varvec{u}_h)&\leqslant \Vert \varvec{g} \Vert _{L^\infty (\varOmega )} \Vert \varrho _h \Vert \Vert \varvec{u}_h \Vert \leqslant \Vert \varvec{g} \Vert _{L^\infty (\varOmega )} \Vert \varrho _h \Vert \Vert (\varvec{u}_h, {\widehat{ \varvec{u}}}_h) \Vert _{1,h}, \end{aligned}$$

thus we conclude $\nu \Vert (\varvec{u}_h, {\widehat{ \varvec{u}}}_h) \Vert _{1,h} \lesssim \Vert \varvec{f} \Vert + \Vert \varvec{g} \Vert _{L^\infty (\varOmega )} \Vert \varrho _h \Vert$. Note, that by the above construction we have also proven (13).

For the proof of (14) let $p_h:= p(\varrho _h)$ and define the mean value ${\overline{p}}_h:= | \varOmega |^{-1} \int _\varOmega p_h$. By Lemma 1 it exists a $\varvec{v}_h \in \varvec{V}_h$ with $\textrm{div} \varvec{v}_h = p_h - {\overline{p}}_h$ such that

$$\begin{aligned} \Vert p_h - {\overline{p}}_h \Vert ^2 = b(p_h - {\overline{p}}_h, \varvec{v}_h), \end{aligned}$$

and $$\Vert (\varvec{v}_h, \widehat{\varvec{v}}_h) \Vert _{1,h}\lesssim \Vert p_h - \overline{p}_h \Vert $$. Hence

$$\begin{aligned} \Vert p_h - \overline{p}_h \Vert ^2&=b(p_h - {\overline{p}}_h, \varvec{v}_h) \\&= -a_h((\varvec{u}_h, \widehat{\varvec{u}}_h),(\varvec{v}_h, \widehat{\varvec{v}}_h)) + F_h(\varvec{v}_h) + G_h(\varrho _h, \varvec{v}_h)\\&\lesssim \nu \Vert (\varvec{u}_h, \widehat{\varvec{u}}_h ) \Vert _{1,h} \Vert (\varvec{v}_h, \widehat{\varvec{v}}_h ) \Vert _{1,h} + \Vert \varvec{f} \Vert \Vert \varvec{v}_h \Vert + |G_h(\varrho _h, \varvec{v}_h) |\\&\lesssim \left( \Vert \varvec{f} \Vert + \Vert \varvec{g} \Vert _{L^\infty (\varOmega )} \Vert \varrho _h \Vert \right) \Vert p_h - \overline{p}_h \Vert . \end{aligned}$$

where we again used a discrete Friedrichs inequality in the last step.

The mass constraint (8c) yields the identity

$$\begin{aligned} \overline{p}_h = \frac{1}{|\varOmega |} \int _\varOmega p_h = \frac{1}{|\varOmega |} \int _\varOmega {c_M}\varrho _h = {c_M}M |\varOmega |^{-1}, \quad \text {thus} \quad \Vert \overline{p}_h \Vert ^2 = {c_M}^2 M^2 |\varOmega |^{-1}. \end{aligned}$$

Eventually, a Pythagoras theorem and the previous estimates yield

$$\begin{aligned} {c_M}^2 \Vert \varrho _h \Vert ^2 = \Vert p_h \Vert ^2&= \Vert p_h-{\overline{p}}_h \Vert ^2 + \Vert {\overline{p}}_h \Vert ^2\\&\leqslant C \left( \Vert \varvec{f} \Vert ^2 + \Vert \varvec{g} \Vert _{L^\infty (\varOmega )} ^2 \Vert \varrho _h \Vert ^2 \right) + {c_M}^2 M^2 |\varOmega |^{-1}, \end{aligned}$$

which can be reordered into

$$\begin{aligned} \left( 1 - C {c_M}^{-2} \Vert \varvec{g} \Vert _{L^\infty (\varOmega )} ^2 \right) \Vert \varrho _h \Vert ^2 \lesssim {c_M}^{-2}\Vert \varvec{f} \Vert ^2 + M^2. \end{aligned}$$

This concludes the proof. $\square$

### Existence of Discrete Solutions

This section suggests a fixed-point iteration for the computation of a solution of (8) and shows existence of at least one fixed-point. The steps are very similar to [1]. Throughout this section we assume the lowest order case $k=1$ to guarantee that all computed densities stay non-negative.

#### Algorithm 2

(Fixed-point algorithm) Given a triangulation $\mathcal {T}$ and a step size $\tau > 0$ and initial values $\varvec{u}_h^0 = \varvec{0}$ and $\varrho _h^0:= M / |\varOmega |$, compute, for $n = 0,1,2,\ldots$ until satisfied,

$$\begin{aligned} (\varvec{u}^{n+1}_h, \widehat{\varvec{u}}^{n+1}_h, \varrho ^{n+1}_h) = F((\varvec{u}^{n}_h, \widehat{\varvec{u}}^{n}_h, \varrho ^{n}_h)), \end{aligned}$$

where $F: \varvec{V}_h \times \widehat{\varvec{V}}_h \times Q_h \rightarrow \varvec{V}_h \times \widehat{\varvec{V}}_h \times Q_h$ denotes the fixed-point mapping that computes the new iterate by the following sub-systems. The new velocity iterate $(\varvec{u}^{n+1}_h, \widehat{\varvec{u}}^{n+1}_h) \in \varvec{V}_h \times \widehat{\varvec{V}}_h$ satisfies, for all $(\varvec{v}_h, \widehat{\varvec{v}}_h) \in \varvec{V}_h \times \widehat{\varvec{V}}_h$,

16 $$\begin{aligned} \nu a_h((\varvec{u}^{n+1}_h, \widehat{\varvec{u}}^{n+1}_h),(\varvec{v}_h, \widehat{\varvec{v}}_h))&= F_h(\varvec{v}_h) + G_h(\varrho _h^{n}, \varvec{v}_h) - b(p(\varrho ^{n}_h), \varvec{v}_h), \end{aligned}$$

and the new density iterate $\varrho _h^{n+1} \in Q_h$ satisfies

17 $$\begin{aligned} \tau ^{-1} (\varrho _h^{n+1}, \lambda _h) + c_h(\varrho ^{n+1}_h, \varvec{u}^{n+1}_h, \lambda _h) = \tau ^{-1} (\varrho ^{n}_h, \lambda _h) \quad \text {for all } \lambda _h \in Q_h. \end{aligned}$$

The iteration is stopped if the residuals of both sub-systems are below some given tolerance.

#### Lemma 4

(Solvability of the sub-systems) Both sub-systems (16) and (17) are solvable. Moreover, if $\rho _h^{n} > 0$ and $(\rho ^n_h,1) = M$, then also $\rho _h^{n+1} > 0$ and $(\rho ^{n+1}_h,1) = M$.

#### Proof

The solvability of the update (16) for $\varvec{u}^{n+1}_h$ follows from the coercivity of $a_h$, see (15).

The solvability of the update (17) for $\varrho ^{n+1}_h$ follows from the fact that for $k=1$ (i.e. $\varrho$ is approximated by piecewise constants) the system matrix is an *M*-matrix. Indeed, the representation matrix for the form $c_h(\varrho _h^{n+1}, \varvec{u}_h^{n+1}, \lambda _h)$ (for fixed $\varvec{u}_h^{n+1})$ is weakly diagonal-dominant, has non-negative diagonal entries and non-positive off-diagonal entries, and has zero row-sums. Hence, adding a positive definite diagonal matrix yields an *M*-matrix. That matrix is invertible and has only positive entries. Hence, the positivity of the previous density iterate $\varrho _h^n$ is preserved. Moreover, also the mass constraint is preserved which follows from testing with $\lambda _h \equiv 1$. $\square$

The following lemma establishes existence of a fixed-point via Brouwer’s fixed-point theorem.

#### Lemma 5

(Existence of solutions) On every fixed shape-regular mesh $\mathcal {T}$, the discrete nonlinear system (8) has at least one solution.

#### Proof

The mapping *F* that defines the fixed-point iteration in Algorithm 2 is linear and continuous, since it consists of the composition of two solvable linear systems of equations, see Lemma 4.

To apply Brouwer’s fixed-point theorem, it remains to show that *F* maps a convex set into itself. This can be shown by similar arguments as in Theorem 1, but the term $b(p(\varrho ^{n}_h), \varvec{v}_h)$ has to be estimated by

$$\begin{aligned} b(p(\varrho ^{n}_h), \varvec{v}_h) \leqslant \Vert \textrm{div} (\varvec{v}_h) \Vert \Vert p(\varrho ^{n}_h) \Vert \leqslant {c_M}\Vert (\varvec{v}_h, {\widehat{\varvec{v}}}_h) \Vert _{1,h} \Vert \varrho ^{n}_h \Vert . \end{aligned}$$

Since all discrete norms on the fixed triangulation $\mathcal {T}$ are equivalent (with some possibly mesh-dependent constant *C*(*h*)) and the mass constraint is preserved in every iteration, we can employ the pessimistic but sufficient bound

$$\begin{aligned} \Vert \varrho ^{n}_h \Vert \leqslant C(h) \Vert \varrho ^{n}_h \Vert _{L^1} = C(h) M. \end{aligned}$$

Hence, all iterates stay within a bounded convex set, which justifies the application of Brouwer’s fixed-point theorem to conclude the existence of a fixed-point. $\square$

## Convergence of the Scheme

This section shows convergence of the discrete solutions to a weak solution of the model problem under suitable assumptions.

For this we apply a Rellich-type theorem of [22] for (H)DG approximations. Although [22] considers only the scalar case, the vector valued case follows accordingly. The result involves an element-wise lifting operator, defined on each $T \in \mathcal {T}$ by

$$\begin{aligned} R_h|_T : \varvec{L}^2(\partial T) \rightarrow [P_k(T)]^{d \times d}, \quad \varvec{g} \mapsto \varPhi _h, \end{aligned}$$

where $\varPhi _h$ is given by

$$\begin{aligned} (\varPhi _h, \varPsi _h)_T = (\varvec{g}, \varPsi _h \varvec{n})_{\partial T} \quad \text {for all } \quad \varPsi _h \in [P_k(T)]^{d \times d}. \end{aligned}$$

Moreover, there is the operator $S_h: \varvec{H}^1(\mathcal {T}) \rightarrow \prod _{T \in \mathcal {T}} \varvec{L}^2(\partial T)$ defined by

$$\begin{aligned} S_h (\varvec{u}) := \bigl \lbrace \left( \varvec{u}|_T\right) |_{\partial T} \bigr \rbrace _{T \in \mathcal {T}}, \end{aligned}$$

that collects all cell boundary traces.

### Theorem 3

Consider a sequence of shape-regular triangulations $(\mathcal {T}_h)_{h \rightarrow 0}$. Let $((\varvec{u}_h, \widehat{\varvec{u}}_h), \varrho _h)$ denote the corresponding discrete solution of (8) on $\mathcal {T}_h$. Then, up to extraction of a subsequence, it holds

(i) the sequence $(\varvec{u}_h)_{h \rightarrow 0}$ converges strongly to some $\varvec{u} \in \varvec{L}^2(\varOmega ) \cap \varvec{H}^1_0(\varOmega )$ and $\nabla \varvec{u}_h + R_h(\widehat{\varvec{u}}_h + (S_h \varvec{u}_h)_t) \rightharpoonup \nabla \varvec{u}$,
(ii) the sequence $(\varrho _h)_{h \rightarrow 0}$ converges weakly in $L^2(\varOmega )$ to a limit $\varrho \in L^2(\varOmega )$,
(iii) the sequence $(p_h)_{h \rightarrow 0}:= (p(\varrho _h))_{h \rightarrow 0}$ converges weakly in $L^2(\varOmega )$ to a limit $p_\star \in L^2(\varOmega )$,
(iv) $p_\star$ and $\varrho$ satisfy the equation of state, i.e., $p_\star = p(\varrho )$,
(v) the limit $(\varvec{u}, \varrho )$ is a weak solution of (3).

### Proof of (i)-(iii)

By Theorem 1 the sequence $\varvec{u}_h$ is bounded and the result follows from [22, Theorem 1]. $\square$

### Proof of (iv)

For $\gamma = 1$ this is straightforward, since the equation of state is linear. To see this consider a function $\varphi \in C_c^\infty (\varOmega )$ and some sequence $\varphi _h:= \varPi _{Q_h} \varphi$ that converges strongly towards $\varphi$. For that sequence, due to weak-strong convergence, it holds

$$\begin{aligned} (p_h, \varphi _k)&\rightarrow (p_\star , \varphi ), \end{aligned}$$

and on the other hand

$$\begin{aligned} (p_h, \varphi _k) = (p(\varrho _k), \varphi _k) = (c \varrho _k, \varphi _k)&\rightarrow (c \varrho , \varphi ) = (p(\varrho ), \varphi ). \end{aligned}$$

This allows to conclude

$$\begin{aligned} (p_\star - p(\varrho ), \varphi ) = 0 \quad \text {for all } C_c^\infty (\varOmega ), \end{aligned}$$

which implies (iv). $\square$

### Proof of (v)

We first prove that $(\varvec{u}, \varrho )$ satisfy the momentum equation (3a). Take any vector-valued smooth test function $\varvec{v} \in \varvec{C}_c^\infty (\varOmega )$ and approximate it by best-approximations $\varvec{v}_h \in \varvec{V}_h \! \cap \! \varvec{H}^1_0(\varOmega )$ such that

$$\begin{aligned} \varvec{v}_h \rightarrow \varvec{v} \quad \text {strong in } \varvec{H}_0^1(\varOmega ). \end{aligned}$$

Now choose $\widehat{\varvec{v}}_h = (\varvec{v}_h)_t$ then strong-weak convergence, see [22], yields

$$\begin{aligned} a_h((\varvec{u}_h, \widehat{\varvec{u}}_h),(\varvec{v}_h, \widehat{\varvec{v}}_h))&:= \sum _{T \in \mathcal {T}} (\nabla \varvec{u}_h, \nabla \varvec{v}_h)_T + (\nabla \varvec{v}_h \varvec{n}, (\widehat{\varvec{u}}_h - \varvec{u}_h)_t)_{\partial T} \\&= \sum _{T \in \mathcal {T}} (\nabla \varvec{u}_h + R_h|_T((\widehat{\varvec{u}}_h - S_h \varvec{u}_h)_t), \nabla \varvec{v}_h)_T\\&\rightarrow (\nabla \varvec{u}, \nabla \varvec{v}) = a(\varvec{u},\varvec{v}). \end{aligned}$$

Next, since $\varvec{v}_h$ is continuous, we have

$$\begin{aligned} b_h(p(\varrho _h), \varvec{v}_h)&= \sum _{T \in \mathcal {T}} - (\textrm{div}(\varvec{v}_h), p(\varrho _h))_T = -\int _\varOmega \textrm{div}(\varvec{v}_h) p(\varrho _h) \rightarrow -\int _\varOmega \textrm{div}(\varvec{v}) p(\varrho ). \end{aligned}$$

Using strong convergence and weak-strong convergence one also obtains

$$\begin{aligned} \int _\varOmega \varvec{f} \varvec{v}_h \rightarrow \int _\varOmega \varvec{f} \varvec{v}, \quad \text {and} \quad \int _\varOmega \varvec{g} \varrho _h \varvec{v}_h \rightarrow \int _\varOmega \varvec{g} \varrho \varvec{v}. \end{aligned}$$

It remains to prove that the limits fulfill the continuity equation. Consider a test function $\psi \in \varvec{C}^\infty (\varOmega )$ and approximate it by best-approximations $\psi _h \in P_1(\mathcal {T}) \cap \varvec{H}^1(\varOmega )$ such that

$$\begin{aligned} \psi _h \rightarrow \psi \quad \text {strong in } \varvec{H}^1(\varOmega ) \quad \text {and} \quad \Vert \nabla \psi _h \Vert _{L^\infty (\varOmega )} \lesssim \Vert \nabla \psi \Vert _{L^\infty (\varOmega )} . \end{aligned}$$

The bound follows from an inverse inequality for polynomials and the stability of the $H^1$-best-approximation, i.e.

$$\begin{aligned} \Vert \nabla \psi _h \Vert _{L^\infty (\varOmega )} \lesssim h^{-d/2} \Vert \nabla \psi _h \Vert \lesssim h^{-d/2} \Vert \nabla \psi \Vert , \end{aligned}$$

and the estimate

$$\begin{aligned} \Vert \nabla \psi \Vert ^2 \leqslant \Vert \nabla \psi \Vert _{L^1} \Vert \nabla \psi \Vert _{L^\infty (\varOmega )} \leqslant \Vert 1\Vert \Vert \nabla \psi \Vert \Vert \nabla \psi \Vert _{L^\infty (\varOmega )} \approx h^{d/2} \Vert \nabla \psi \Vert \Vert \nabla \psi \Vert _{L^\infty (\varOmega )} . \end{aligned}$$

The discrete momentum $\varrho _h \varvec{u}_h$ is approximated into some $q_h \in \varvec{V}_h \subset \varvec{H}(\textrm{div}, \varOmega )$ by

18 $$\begin{aligned} \varvec{q}_h|_T := I_h^{\textrm{RT}_0} (\varrho ^{\text {up}}_h \varvec{u}_h|_T) \quad \text {on each } T \in \mathcal {T}, \end{aligned}$$

where $I_h^{\textrm{RT}_0}$ is the interpolation operator into the lowest order Raviart–Thomas space, see [4]. Note, that this interpolation is divergence-free, because $\textrm{div}(\varvec{q}_h|_T){} \in P_0(\mathcal {T})$ and

$$\begin{aligned} (\textrm{div}(\varvec{q}_h|_T), \chi _T) = c_h(\varrho _h, \varvec{u}_h, \chi _T) = 0. \end{aligned}$$

Here, $\chi _T \in Q_h$ is the indicator function of *T*, i.e. $\chi _T = 1$ on *T* and zero elsewhere. With that, it holds

$$\begin{aligned} 0 = (\psi _h, \textrm{div} \, \varvec{q}_h) = - (\varvec{q}_h, \nabla \psi _h) = -( \varvec{q}_h - \varrho _h \varvec{u}_h, \nabla \psi _h) - (\varrho _h \varvec{u}_h, \nabla \psi _h). \end{aligned}$$

It remains to show that the first term on the right-hand side converges to zero. A triangle inequality yields

19 $$\begin{aligned} \begin{aligned}&| ( \varvec{q}_h - \varrho _h \varvec{u}_h, \nabla \psi _h) | \\&\quad \leqslant \sum _{T \in \mathcal {T}} \left| \nabla \psi _h|_T \cdot \left( \int _T (\varvec{q}_h - \varrho _h I_h^{\textrm{RT}_0} \varvec{u}_h) \, + \int _T \varrho _h (I_h^{\textrm{RT}_0} \varvec{u}_h - \varvec{u}_h) \, \right) \right| \\&\quad \leqslant C \sum _{T \in \mathcal {T}} \Vert \varvec{q}_h - \varrho _h I_h^{\textrm{RT}_0} \varvec{u}_h \Vert _{L^1(T)} + C \Vert \varrho _h \Vert \, \Vert I_h^{\textrm{RT}_0} \varvec{u}_h - \varvec{u}_h \Vert . \end{aligned} \end{aligned}$$

The term $\Vert \varrho _h \Vert \, \Vert I_h^{\textrm{RT}_0} \varvec{u}_h - \varvec{u}_h \Vert$ converges to 0, according to the interpolation properties of $I_h^{\textrm{RT}_0}$ and the stability estimate for $\Vert \varrho _h \Vert$ and $\Vert (\varvec{u}_h, \widehat{\varvec{u}}_h) \Vert _{1,h}$. It remains to estimate $\sum _T \Vert \varvec{q}_h - \varrho _h I_h^{\textrm{RT}_0} \varvec{u}_h \Vert _{L^1(T)}$. Interpolation properties of $I_h^{\textrm{RT}_0}$ yield

$$\begin{aligned} \sum _T \Vert \varvec{q}_h - \varrho _h I_h^{\textrm{RT}_0} \varvec{u}_h \Vert _{L^1(T)}&\lesssim \sum _T h_T \sum _{F \in \mathcal {F}(T)} \left|(\varrho ^\text {up}_h - \varrho _h|_T ) \int _F \varvec{u}_h \cdot \varvec{n}_F \, ds \right|\\&\lesssim \sum _{F \in \mathcal {F}(\varOmega )} h_F |[\![{ \varrho _h}]\!]_F |\, \left|\int _F \varvec{u}_h \cdot \varvec{n}_F \right|:= A. \end{aligned}$$

There holds $A \rightarrow 0$ which can be proven as follows. A Cauchy inequality shows

$$\begin{aligned} A \lesssim \left( \sum _{F \in \mathcal {F}(\varOmega )} \left|\int _F \varvec{u}_h \cdot \varvec{n}_F \right|\left( \varrho _h^F\right) ^{-1} [\![{ \varrho _h}]\!]^2_F \right) ^{1/2} \left( \sum _{F \in \mathcal {F}(\varOmega )} h_F^2 \left|\int _F \varvec{u}_h \cdot \varvec{n}_F \right|\varrho _h^F \right) ^{1/2}. \end{aligned}$$

The left sum is bounded by Theorem 1. To show that the second sum converges to zero, we employ a Hölder inequality, a trace inequality and an inverse inequality on some neighboring simplex $T_F$ of $F$ to obtain

20 $$\begin{aligned} \left|\int _F \varvec{u}_h \cdot \varvec{n}_F \right|\lesssim \Vert \varvec{u}_h \Vert ^{1/2}_{T_F} \Vert \nabla \varvec{u}_h \Vert ^{1/2}_{T_F} \Vert 1 \Vert _{F} \lesssim h_F^{(d-2)/2} \Vert \varvec{u}_h \Vert _{T_F}. \end{aligned}$$

Hence,

$$\begin{aligned} \left( \sum _{F \in \mathcal {F}(\varOmega )} h_F^2 \left|\int _F \varvec{u}_h \cdot \varvec{n}_F \right|\varrho _h^F \right) ^{1/2} \lesssim \left( \sum _{F \in \mathcal {F}(\varOmega )} h_F^{(d+2)/2} \Vert \varvec{u}_h \Vert _{T_F} \varrho _h^F\right) ^{1/2}. \end{aligned}$$

Then, another Cauchy inequality, a Friedrichs inequality for piecewise $H^1$ functions [6] and some overlap arguments yield

$$\begin{aligned} \left( \sum _{F \in \mathcal {F}(\varOmega )} h_F^2 \left|\int _F \varvec{u}_h \cdot \varvec{n}_F \right|\varrho _h^F \right) ^{1/2}&\leqslant \left( \sum _{F \in \mathcal {F}(\varOmega )} \Vert \varvec{u}_h\Vert _{T_F}^2 \right) ^{1/4} \!\! \left( \sum _{F \in \mathcal {F}(\varOmega )} h_F^{d+2} \left( \varrho _h^F\right) ^2 \right) ^{1/4}\\&\lesssim \Vert (\varvec{u}_h, {\widehat{\varvec{u}}}_h) \Vert ^{1/2}_{1,h} \left( \sum _{F \in \mathcal {F}(\varOmega )} h_F^{d+2} \left( \varrho _h^F\right) ^2 \right) ^{1/4}. \end{aligned}$$

Since $\varrho _h^F$ is smaller than $\varrho _h|_{T_F}$ for some neighboring simplex $T_F$ of $T$, we also can bound the remaining sum by

$$\begin{aligned} \left( \sum _{F \in \mathcal {F}(\varOmega )} h_F^{d+2} \left( \varrho _h^F\right) ^2 \right) ^{1/4} \lesssim \left( \sum _{F \in \mathcal {F}(\varOmega )} h_F^2 |T_F |\varrho _h|_{T_F}^2 \right) ^{1/4} \leqslant h^{1/2} \Vert \varrho _h \Vert ^{1/2}. \end{aligned}$$

According to Theorem 1 the norm $\Vert (\varvec{u}_h, {\widehat{\varvec{u}}}_h) \Vert _{1,h}$ and $\Vert \varrho _h \Vert$ are bounded and so we eventually arrive at

$$\begin{aligned} A \lesssim h^{1/2}. \end{aligned}$$

This and weak-strong convergence ($\varvec{u}_h \nabla \psi _h$ converges strongly against $\varvec{u} \nabla \psi$) implies

$$\begin{aligned} |(\varrho \varvec{u}, \nabla \psi ) |&= |(\varrho \varvec{u}, \nabla \psi ) - (\varrho _h \varvec{u}_h, \nabla \psi _h) |+ |(\varrho _h \varvec{u}_h, \nabla \psi _h) |\\&\lesssim |(\varrho \varvec{u}, \nabla \psi ) - (\varrho _h \varvec{u}_h, \nabla \psi _h) |+ h^{1/2} \rightarrow 0. \end{aligned}$$

This concludes the proof. $\square$

### Remark 2

(Asmyptotic convergence to a pressure-robust scheme) On a fixed mesh and for ${c_M}\rightarrow \infty$, the solutions of the scheme 8 converge to a pressure-robust divergence-free solution of the incompressible Stokes equations, see [1, Lemma 6.4] for details and a proof.

## A Fully Discontinuous HDG Scheme

This section elaborates on the qualitative improvements by strictly enforcing the normal continuity of the velocity. As for the Stokes model problem, the $H(\textrm{div})$ conformity yields $L^2$ orthogonality of the divergence-free part of the velocity with gradients. This property is lost when a full HDG scheme is used that also allows jumps of the normal component. For comparison in the numerical experiments also this scheme shall be briefly discussed.

The ansatz spaces for this variant reads as

$$\begin{aligned} \varvec{V}_h&:= \varvec{P}_{k}(\mathcal {T}),\\ \widehat{\varvec{V}}_h&:= \left\{ \widehat{\varvec{v}}_h \in \varvec{L}^2(\mathcal {F}): \widehat{\varvec{v}}_h|_F \in \varvec{P}_{k}(F), \widehat{\varvec{v}}_h = 0 \text { on } \partial \varOmega \right\} ,\\ Q_h&:= P_{k-1}(\mathcal {T}). \end{aligned}$$

The full HDG scheme seeks $\left( (\varvec{u}_h, \widehat{\varvec{u}}_h), (\varrho _h, {\widehat{\varrho }})\right) \in (\varvec{V}_h \times \widehat{\varvec{V}}_h) \times Q_h$ such that

21a $$\begin{aligned} \nu a_h((\varvec{u}_h, \widehat{\varvec{u}}_h),(\varvec{v}_h, \widehat{\varvec{v}}_h)) + b_h(p(\varrho _h), \varvec{v}_h)&= F_h(\varvec{v}_h) + G_h(\varrho _h, \varvec{v}_h), \end{aligned}$$

21b $$\begin{aligned} c_h(\varrho _h, \varvec{u}_h, \lambda _h)&= 0, \end{aligned}$$

21c $$\begin{aligned} (\varrho _h,1)&= M, \end{aligned}$$

for all $(\varvec{v}, \widehat{\varvec{v}}_h) \in \varvec{V}_h \times \widehat{\varvec{V}}_h$ and $\lambda _h \in Q_h$. Here, the forms are defined by

$$\begin{aligned} a_h((\varvec{u}_h, \widehat{\varvec{u}}_h),(\varvec{v}_h, \widehat{\varvec{v}}_h))&:= \sum _{T \in \mathcal {T}} (\nabla \varvec{u}_h, \nabla \varvec{v}_h)_T + (\nabla \varvec{u}_h \varvec{n}, (\widehat{\varvec{v}}_h - \varvec{v}_h))_{\partial T}\\&\quad + (\nabla \varvec{v}_h \varvec{n}, (\widehat{\varvec{u}}_h - \varvec{u}_h))_{\partial T} + \frac{\alpha k^2}{h} ((\widehat{\varvec{u}}_h - \varvec{u}_h), (\widehat{\varvec{v}}_h - \varvec{v}_h))_{\partial T},\\ b_h(\varrho _h, (\varvec{v}_h, {\widehat{\varvec{v}}}_h))&:= \sum _{T \in \mathcal {T}} -(\varrho _h, \textrm{div} \varvec{v}_h)_T + ((\varvec{v}_h - {\widehat{ \varvec{v}}}_h) \cdot \varvec{n}, \varrho _h)_{\partial T},\\ c_h(\varrho _h, (\varvec{u}_h,{\widehat{\varvec{u}}}_h), \lambda _h)&:=-\sum _{T \in \mathcal {T}} (\varrho _h \varvec{u}_h, \nabla \lambda _h)_T + ({\widehat{\varvec{u}}}_h \cdot \varvec{n} \varrho _h^{up}, \lambda _h)_{\partial T}, \\ G_h(\varrho _h, \varvec{v}_h)&:= (\varvec{g}, \varrho _h \varvec{v}_h),\\ F_h(\varvec{v}_h)&:= (\varvec{f}, \varvec{v}_h). \end{aligned}$$

Compared to (8) the missing normal-continuity causes some changes. In particular, the upwinding term in $c_h$ now involves ${\widehat{\varvec{u}}}_h \cdot \varvec{n}$, which can be interpreted as a mean value of the potentially discontinuous flux $\varvec{u}_h \cdot \varvec{n}$.

Stability and convergence of the scheme can be shown in a similar way as for the $H(\textrm{div})$-conforming HDG scheme (8). Therefore, we only summarize the result and state the main differences in the proof. Note, that the HDG-norm now changes to

$$\begin{aligned} \Vert (\varvec{u}_h, {\widehat{\varvec{u}}}_h) \Vert ^2_{1,h}&:= \sum \limits _{T \in \mathcal {T}} \Vert \nabla \varvec{u}_h \Vert _T^2 + \frac{1}{h_T} \Vert (\varvec{u}_h - {\widehat{\varvec{u}}}_h) \Vert _{\partial T}^2, \end{aligned}$$

but we use the same symbol for simplicity.

### Theorem 4

(Stability) For the solution of (21), it holds

$$\begin{aligned} \Vert (\varvec{u}_h, {\widehat{ \varvec{u}}}_h) \Vert _{1,h}&\lesssim \Vert \varvec{f} \Vert + \Vert \varvec{g} \Vert _{L^\infty (\varOmega )} \Vert \varrho _h \Vert ,\\ \sum _{F \in \mathcal {F}} \phi ^{\prime \prime }(\varrho ^{F}_h) \Big | \int _F {\widehat{\varvec{u}}}_h \cdot \varvec{n}_F [\![{\varrho _h}]\!]^2 \Big |&\lesssim (\Vert \varvec{f} \Vert + \Vert \varvec{g} \Vert _{L^\infty (\varOmega )} \Vert \varrho _h \Vert )^2,\\ \Big (1 - \frac{C}{ {c_M}^2} \Vert \varvec{g} \Vert _{L^\infty (\varOmega )} ^2\Big ) \Vert \varrho _h \Vert ^2&\lesssim \frac{1}{ {c_M}^2} \Vert \varvec{f} \Vert ^2 + M^2, \end{aligned}$$

for some generic constant *C* that depends on the shape of the cells, $|\varOmega |$ and $\alpha$, but not on *h*, ${c_M}$, *M* or $\nu$.

### Proof

The main difference for the stability proof compared to the one of Theorem 1 is that Lemma 3 is employed for $\varvec{u} = {\widehat{\varvec{u}}}_h$ and $\phi (s) = {c_M}s \log (s)$ which yields

$$\begin{aligned} c_h(\varrho _h, \varvec{u}_h, {c_M}(1 + \log (\varrho _h))) + b_h(\varrho _h, (\varvec{u}_h, {\widehat{\varvec{u}}}_h)) = \frac{1}{2} \sum _{F \in \mathcal {F}} \phi ^{\prime \prime }(\varrho ^{F}_h) \Big | \int _F {\widehat{\varvec{u}}}_h \cdot \varvec{n}_F [\![{\varrho _h}]\!]^2 \Big |. \end{aligned}$$

The rest of the arguments is identical. $\square$

### Theorem 5

Consider a sequence of shape-regular triangulations $(\mathcal {T}_h)_{h \rightarrow 0}$. Let $(\varvec{u}_h, \widehat{\varvec{u}}_h, \varrho _h)$ denote the corresponding discrete solution of (21) on $\mathcal {T}_h$. Then, up to extraction of a subsequence, it holds

(i) the sequence $(\varvec{u}_h)_{h \rightarrow 0}$ converges strongly to some $\varvec{u} \in \varvec{L}^2(\varOmega ) \cap \varvec{H}^1_0(\varOmega )$ and $\nabla \varvec{u}_h + R_h(\widehat{\varvec{u}}_h + S_h \varvec{u}_h) \rightharpoonup \nabla \varvec{u}$,
(ii) the sequence $(\varrho _h)_{h \rightarrow 0}$ converges weakly in $L^2(\varOmega )$ to a limit $\varrho \in L^2(\varOmega )$,
(iii) the sequence $(p_h)_{h \rightarrow 0}:= (p(\varrho _h))_{h \rightarrow 0}$ converges weakly in $L^2(\varOmega )$ to a limit $p_\star \in L^2(\varOmega )$,
(iv) $p_\star$ and $\varrho$ satisfy the equation of state, i.e., $p_\star = p(\varrho )$,
(v) the limit $(\varvec{u}, \varrho )$ is a weak solution of (3).

### Proof

Again, only the main differences compared to the proof of Theorem 3 are stated. First, the choice of (18) has to be altered to

$$\begin{aligned} \varvec{q}_h|_T := I_h^{\textrm{RT}_0} (\varrho _{\text {upw}} \widehat{\varvec{u}}_h|_T) \quad \text {on each } T \in \mathcal {T} \end{aligned}$$

which then again is divergence-free. In the critical estimate (19) one now obtains

$$\begin{aligned} \begin{aligned}&| ( \varvec{q}_h - \varrho _h \varvec{u}_h, \nabla \psi _h) |\\&\quad \leqslant \sum _T \left| \nabla \psi _h|_T \cdot \left( \int _T (\varvec{q}_h - \varrho _h I_h^{\textrm{RT}_0} {\widehat{\varvec{u}}}_h) \, + \int _T \varrho _h (I_h^{\textrm{RT}_0} {\widehat{\varvec{u}}}_h - \varvec{u}_h) \, \right) \right| \\&\quad \leqslant C \sum _T \Vert \varvec{q}_h - \varrho _h I_h^{\textrm{RT}_0} {\widehat{\varvec{u}}}_h \Vert _{L^1(T)} + C \Vert \varrho _h \Vert \, \Vert I_h^{\textrm{RT}_0} {\widehat{\varvec{u}}}_h - \varvec{u}_h \Vert . \end{aligned} \end{aligned}$$

Here, the first term is treated as in the other proof and the second term can be bounded by

$$\begin{aligned} \Vert I_h^{\textrm{RT}_0} {\widehat{\varvec{u}}}_h - \varvec{u}_h \Vert ^2&\lesssim \sum _F h \left( \int _F ({\widehat{\varvec{u}}}_h - \varvec{u}_h) \cdot \varvec{n} ds \right) ^2\\&\lesssim \sum _T h^3 \Vert (\varvec{u}_h - {\widehat{\varvec{u}}}_h) \Vert _{\partial T}^2 \leqslant h^4 \Vert (\varvec{u}_h - {\widehat{\varvec{u}}}_h) \Vert _{1,h}^2. \end{aligned}$$

The rest of the proof is identical. $\square$

### Remark 3

(Non-gradient-robustness) The scheme (21) is in general not gradient-robust. The reason is that the incompressible Stokes subproblem for the divergence-free part of the solution is not pressure-robust, since the integration by parts in the right-hand side of (10) is not possible without additional jump terms due to the relaxed $H(\textrm{div})$-conformity.

## Numerical Examples

This section studies three numerical examples to compare the two variants with respect to the importance of gradient-robustness and experimental convergence rates. All examples were implemented in the finite element library NGSolve (www.ngsolve.org). It should be noted that static condensation was consistently applied to reduce the number of unknowns when addressing the problems outlined in (16). For further discussion on this matter, we direct the reader to, for instance, [26].

### Convergence Rates

In this section we want to discuss and analyze the approximation properties and error convergence rates of our methods. For this consider the gradient field $\nabla \varPsi := (0, -y^2)^T$, and choose $\varrho$ such that it solves the hydrostatic equation (5), i.e.,

22 $$\begin{aligned} {c_M}\nabla \varrho = \varrho \nabla \varPsi \quad \Rightarrow \quad \varrho := e^{-y^{3} / (3 {c_M})} c^{-1}_\varOmega , \quad \text {with} \quad c_\varOmega = \int _\varOmega e^{-y^{3}/(3 {c_M}) }, \end{aligned}$$

which results in a mass constraint $M = 1$. Further let $\zeta = 100 x^2(1-x)^2y^2(1-y)^2$ and $\varvec{u}:= (-\partial _y \zeta , \partial _x \zeta )/\varrho$, then we define the driving forces

$$\begin{aligned} \varvec{g} := \nabla \varPsi , \quad \text {and} \quad \varvec{f} := - \nu \varDelta \varvec{u}. \end{aligned}$$

By construction we then have $\textrm{div}(\varrho \varvec{u}) = \textrm{div}(\partial _y \zeta , \partial _x \zeta ) = 0$, thus $\varrho$ and $\varvec{u}$ are solutions of the equations (3).

In the following we compare

- the normal continuous approximation ${\varvec{u}_h^{div}}$, i.e. the solution of (8), and
- the fully discontinuous approximation ${\varvec{u}_h^{hdg}}$, i.e. the solution of (21).

**Fig. 1:**
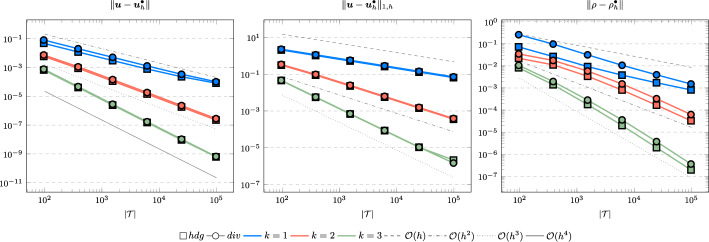
Convergence rates for $k \in \lbrace 1,2,3 \rbrace$ for the unit square test with $\nu = 1$ and ${c_M}= 1$

**Fig. 2:**
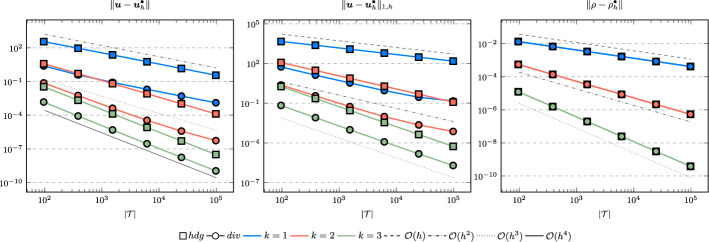
Convergence rates for $k \in \lbrace 1,2,3 \rbrace$ for the unit square test with $\nu = 10^{-6}$ and ${c_M}= 1$

**Fig. 3:**
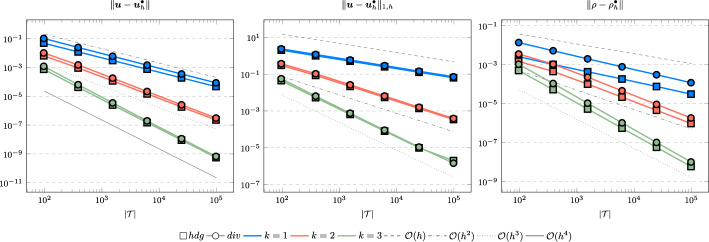
Convergence rates for $k \in \lbrace 1,2,3 \rbrace$ for the unit square test with $\nu = 1$ and ${c_M}= 100$

**Fig. 4:**
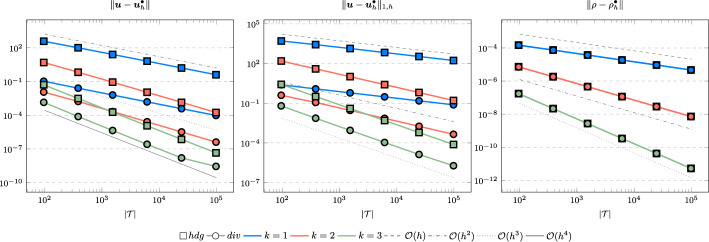
Convergence rates for $k \in \lbrace 1,2,3 \rbrace$ for the unit square test with $\nu = 10^{-6}$ and ${c_M}= 100$

We consider the cases $\nu \in \{1, 10^{-6}\}$ and ${c_M}\in \{1, 100\}$. For all pairs of parameters $(\nu , \varrho )$ Figs. 1, 2, 3, 4 show the convergence history of the $L^2$ error for the velocity and the density as well as the discrete $H^1$ error of the velocity for polynomial orders $k \in \lbrace 1,2,3 \rbrace$. To improve the readability we simplify the notation for the discrete $H^1$ error by

$$\begin{aligned} \Vert \varvec{u} - \varvec{u}_h^\bullet \Vert _{1,h} := \Vert (\varvec{u}, \varvec{u}|_\mathcal {F}) - (\varvec{u}_h^\bullet , \widehat{\varvec{u}}^\bullet _h)\Vert _{1,h}. \end{aligned}$$

We use an initial mesh with 96 elements and a uniform refinement. Note that although we have only proven stability for the lowest order case, the higher order cases were stable for all computations. In the following we discuss the results in more detail:

- The **compressible** case $\nu = 1, {c_M}= 1$, Fig. 1: In this case we do not expect a big difference between the approximations ${\varvec{u}_h^{hdg}}$ and ${\varvec{u}_h^{div}}$. Indeed, all errors are close to each other and we observe an optimal convergence rate.
- The **compressible** case $\nu = 10^{-6}, {c_M}= 1$, Fig. 2: As expected, the velocity errors still converge with optimal order but are deteriorated by the small viscosity. Surprisingly, though still being slightly shifted, the normal continuous approximation ${\varvec{u}_h^{div}}$ is less effected. A similar observation is made for the density error. While still converging with optimal order it scales with the viscosity. In contrast to the velocity approximation no difference between ${\varrho _h^{hdg}}$ and ${\varrho _h^{div}}$ can be found.
- The (nearly) **incompressible cases** $\nu = 1, {c_M}= 100$, Figure 3 and $\nu = 10^{-6}, {c_M}= 100$, Figure 4: Since ${c_M}= 100 \Rightarrow M\!a \approx 10^{-4}$, this setting can be considered as nearly incompressible, thus we expect the result to behave as for a discretization of the incompressible Stokes equations. Indeed, while all errors converge with optimal order, only the normal continuous approximation ${\varvec{u}_h^{div}}$ is pressure-robust, i.e. robust with respect to a small viscosity. This is in agreement with the literature regarding exactly divergence-free approximations, see for example [25].

In contrast to the above setting one could also choose the right hand side as

23 $$\begin{aligned} \varvec{g} := \nabla \varPsi - \frac{\nu \varDelta \varvec{u}}{\varrho }, \quad \text {and} \quad \varvec{f} := 0. \end{aligned}$$

**Fig. 5:**
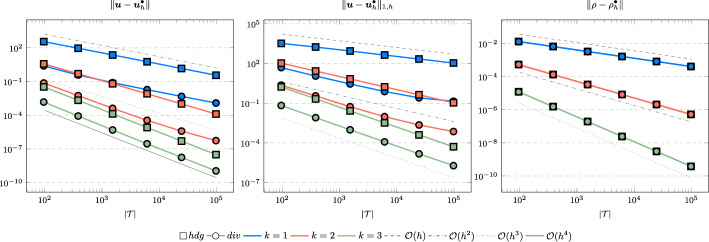
Convergence rates for $k \in \lbrace 1,2,3 \rbrace$ for the unit square test with $\nu = 10^{-6}$ and $c_M = 1$ and right hand side (23)

In Fig. 5 we have plotted the errors for the case ${c_M}= 1$ and $\nu = 10^{-6}$. As one can see (compared to Fig. 2) there is no influence with respect to the choice of the right hand side. For the other cases we observe the same results, thus the plots are omitted for simplicity. Note, that moving the gradient forces $\nabla \varPsi$ also to $\varvec{f}$ results in a big difference, see next example.

### Atmosphere at Rest Over a Mountain

**Fig. 6:**
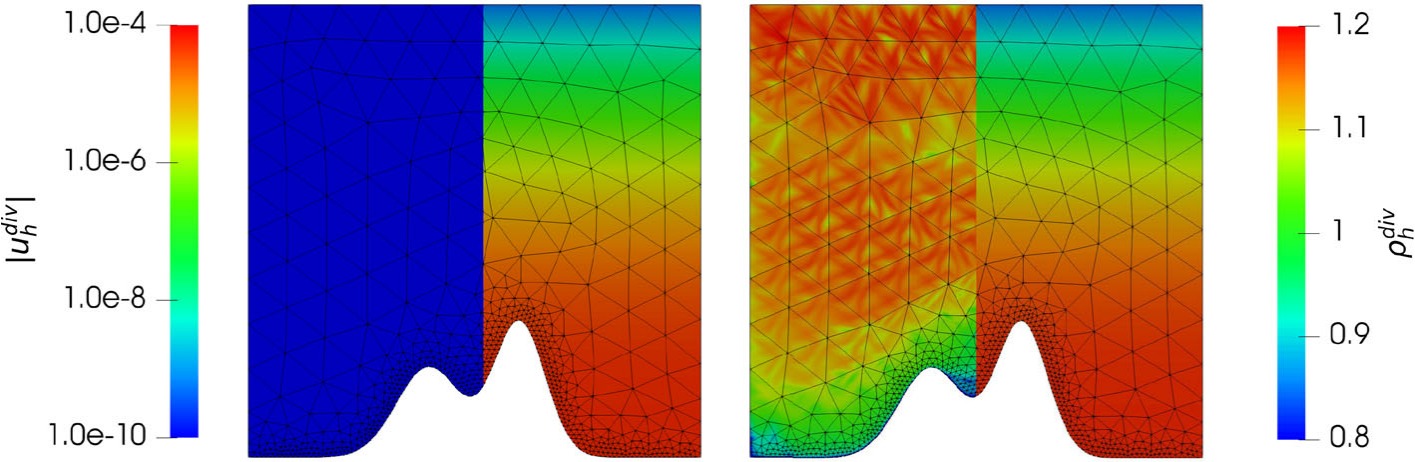
Absolute value of the velocity $| {\varvec{u}_h^{div}}|$ (using a logarithmic scale) plotted on the left side of each plot, and density ${\varrho _h^{div}}$ plotted on the right side of each plot, for $k = 3$ for the mountain example with $\nu = 1$ and $c_M = 1$ (left) and $\nu = 10^{-6}$ and $c_M = 1$ (right)

**Fig. 7:**
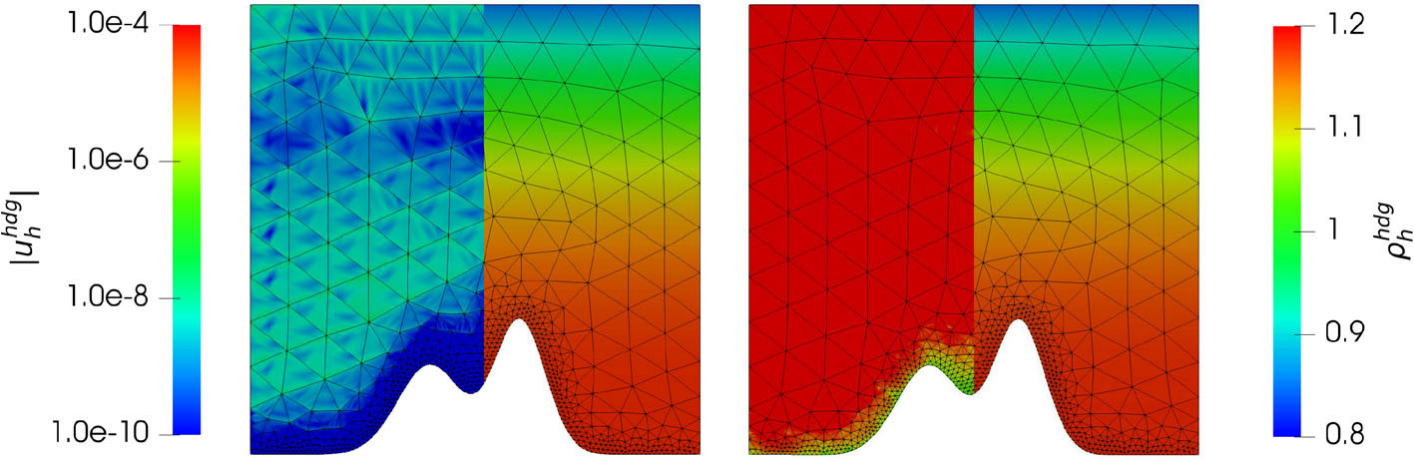
Absolute value of the velocity $| {\varvec{u}_h^{hdg}}|$ (using a logarithmic scale) plotted on the left side of each plot, and density ${\varrho _h^{hdg}}$ plotted on the right side of each plot, for $k = 3$ for the mountain example with $\nu = 1$ and $c_M = 1$ (left) and $\nu = 10^{-6}$ and $c_M = 1$ (right)

**Fig. 8:**
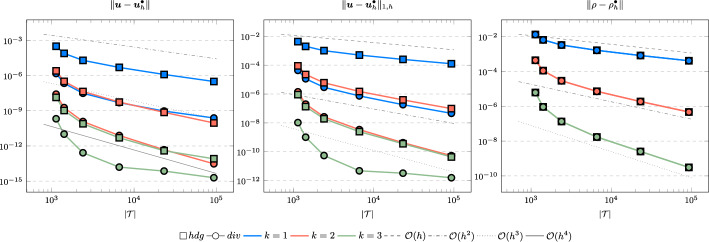
Convergence rates for $k \in \lbrace 1,2,3 \rbrace$ for the mountain example with $\nu = 1$ and $c_M = 1$

**Fig. 9:**
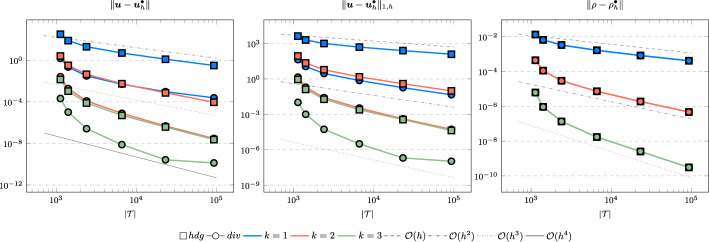
Convergence rates for $k \in \lbrace 1,2,3 \rbrace$ for the mountain example with $\nu = 10^{-6}$ and $c_M = 1$

**Fig. 10:**
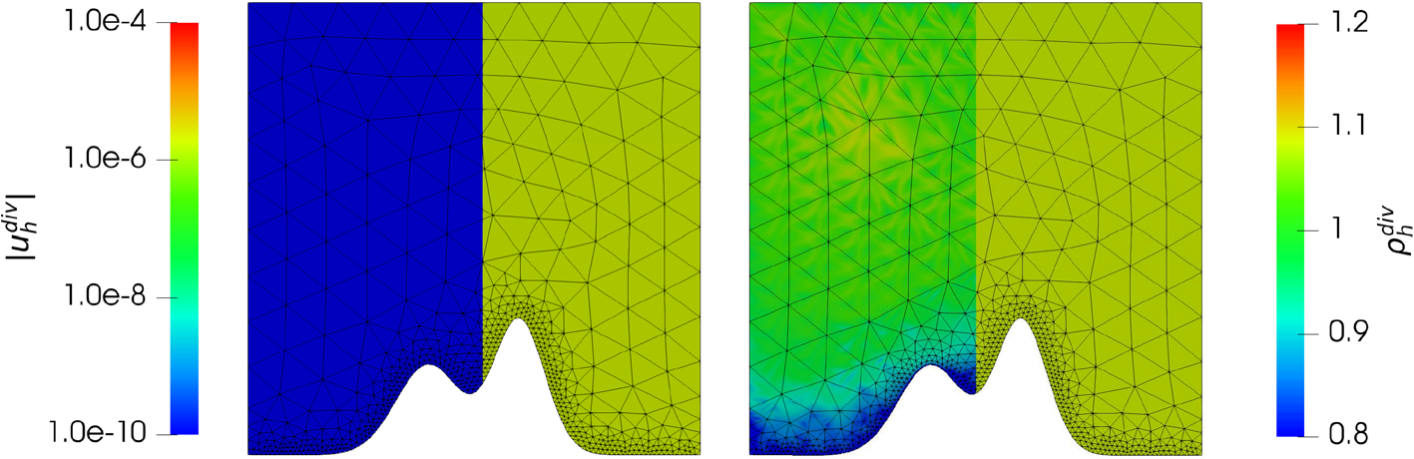
Absolute value of the velocity $| {\varvec{u}_h^{div}}|$ (using a logarithmic scale) plotted on the left side of each plot, and density ${\varrho _h^{div}}$ plotted on the right side of each plot, for $k = 3$ for the mountain example with $\nu = 1$ and $c_M = 100$ (left) and $\nu = 10^{-6}$ and $c_M = 100$ (right)

Consider the *mountain* function given by

$$\begin{aligned} \mathcal {M}(y) := 0.3 e^{\frac{- (y - 0.4)^2}{0.08^2}} + 0.2 e^{\frac{- (y - 0.6)^2}{0.1^2}}, \end{aligned}$$

then we have the domain $\varOmega := \{(x,y) \in \mathbb {R}^2: 0< x< 1, \mathcal {M}(x)< y <1 \}$. We aim to approximate a hydrostatic balance with the exact solution $\varvec{u} = 0$ and $\varrho$ as in the previous example (note that $c_\varOmega$ in (22) changes in order to get $M=1$). This gives $\varvec{g} = \nabla \varPsi = (0, -y^2)^T$. In Fig. 6 and Fig. 7 we have plotted the the absolute value of the velocity ${\varvec{u}_h^{div}}, {\varvec{u}_h^{hdg}}$ and the density ${\varrho _h^{div}},{\varrho _h^{hdg}}$ for $\nu \in \lbrace 1, 10^{-6} \rbrace$ and ${c_M}= 1$ and $k = 3$. Unfortunately, as expected, the velocity error has a dependency on the viscosity $\nu$ similarly as in the previous example, and the normal continuous approximation gives a much better approximation. In Figs. 8 and 9 we give the error history for the mountain example for the same test cases. In contrast to the previous example we did not use nested meshes for the calculation (via a uniform refinement) of errors but used $h_{max} = 0.3 / 2^i$ for $i = 0,\ldots ,5$ for the mesh generator. In addition we used a local mesh size $h_{loc} = 0.01$ in order to properly capture the geometry of the mountain (as can be seen in Figs. 8 and 9). During the mesh generation we used $h_{loc}$ at the mountain surface if $h_{loc} < h_{max}$, and $h_{max}$ otherwise. Thus, although the mesh is initially not quasi uniform, it results in a quasi uniform mesh on later levels. As one can see, this results in an initially fast pre-asymptotic decrease in the error, while the same convergence rate as in the previous example is observed later on. For the highest order case $k = 3$ we see a stagnation of the error close to machine precision. Similarly as in the previous example, for ${c_M}=100$ we only see an improvement for the gradient-robust solution ${\varvec{u}_h^{div}}$, which is plotted in Fig. 10. Also note that one can now clearly see that the density converges to a constant function as expected for a Mach number $M\!a \approx {c_M}^{-2} = 10^{-4}$. We have omitted the results for the fully discontinuous solution ${\varvec{u}_h^{hdg}}$ for ${c_M}= 100$ as the results look similar to Fig. 7 (which is the result for ${c_M}= 1$). In particular the magnitude of the velocity error does not improve with larger ${c_M}$. This is in accordance to the findings of the previous example.

**Fig. 11:**
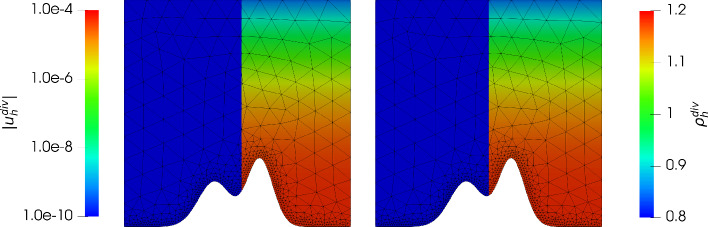
Absolute value of the velocity $|{\varvec{u}_h^{div}}|$ (using a logarithmic scale) plotted on the left side of each plot, and density ${\varrho _h^{div}}$ plotted on the right side of each plot, for $k = 3$ for the mountain example with $\nu = 1$ and $c_M = 1$ (left) and $\nu = 10^{-6}$ and $c_M = 1$ (right) and the right hand side according to (24)

**Fig. 12:**
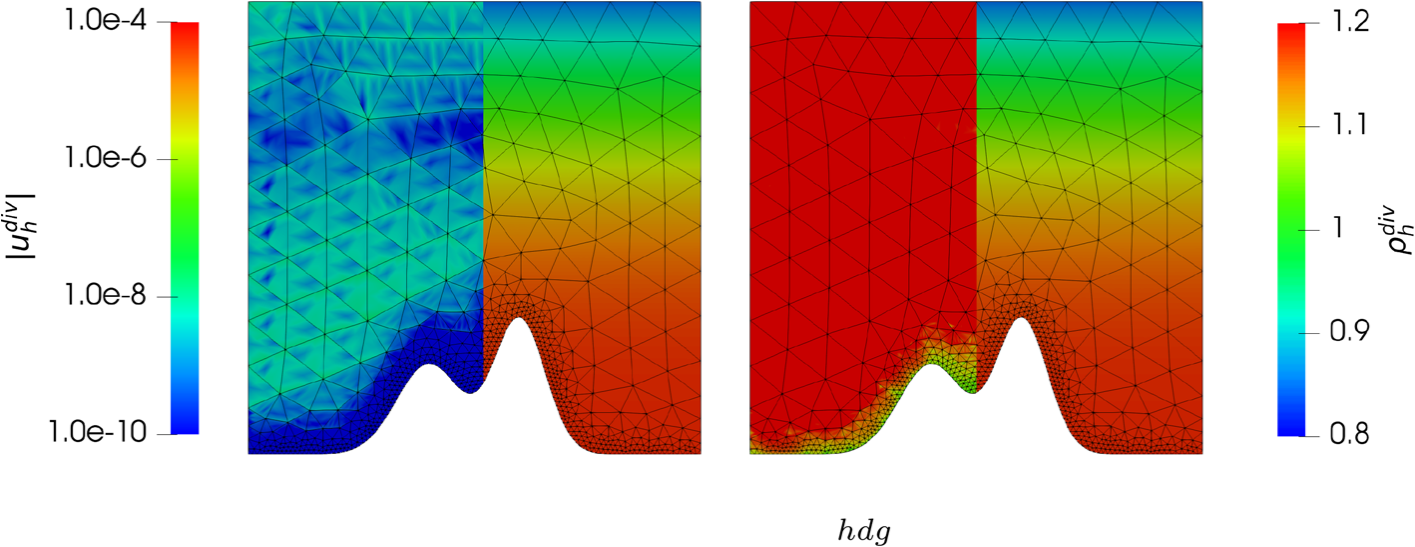
Absolute value of the velocity $|{\varvec{u}_h^{hdg}}|$ (using a logarithmic scale) plotted on the left side of each plot, and density ${\varrho _h^{hdg}}$ plotted on the right side of each plot, for $k = 3$ for the mountain example with $\nu = 1$ and $c_M = 1$ (left) and $\nu = 10^{-6}$ and $c_M = 1$ (right) and the right hand side according to (24)

Finally we present the results if the right hand side is set to

24 $$\begin{aligned} \varvec{g}=0, \quad \text {and} \quad \varvec{f} = \varrho \nabla \varPsi , \end{aligned}$$

still with the same solution after (22). Since $\varvec{f}$ is a gradient field, we expect that the normal continuous solution ${\varvec{u}_h^{div}}$ is not effected by the right hand side for all viscosities due to its gradient-robustness. Indeed, in Figs. 11 and 12 we have again plotted the solutions. As predicted, the solution ${\varvec{u}_h^{div}}$ is always zero (up to machine precision) while the non-pressure robust solution ${\varvec{u}_h^{hdg}}$ is still affected by a decrease of the viscosity and gives the same results as before (compare to Fig. 7).

### A Non-Hydrostatic Well-Balanced State

Gradient-robustness is also relevant, or possibly even more relevant, for the compressible Navier–Stokes problem

25 $$\begin{aligned} - \nu \varDelta \varvec{u} + \varrho (\varvec{u} \cdot \nabla ) \varvec{u} + \nabla p(\varrho ) = 0, \quad \text {and} \quad \textrm{div}(\varrho \varvec{u}) = 0. \end{aligned}$$

Here, the additional convection term balances the pressure gradient at least in the limit $\nu \rightarrow 0$ and non-hydrostatic well-balanced states even in absence of a gravity term are possible. A simple example, for any $\nu \geqslant 0$, can be constructed as follows. Consider the (divergence-free and harmonic) velocity field $\varvec{u}(x,y):= (-y,x)^T$ with the convection term

$$\begin{aligned} \varrho (\varvec{u} \cdot \nabla ) \varvec{u} = - \varrho \begin{pmatrix} x \\ y \end{pmatrix} = - \varrho \frac{1}{2}\nabla \left( x^2 + y^2\right) . \end{aligned}$$

This term can be balanced by the density $\varrho := \varrho _0 \exp ((x^2+y^2)/(2{c_M}))$, since

$$\begin{aligned} \nabla (p(\varrho )) = {c_M}\varrho \nabla (\log \varrho ) = \varrho \frac{1}{2}\nabla \left( x^2 + y^2\right) . \end{aligned}$$

Moreover, the momentum $\varrho \varvec{u}$ is indeed divergence-free due to

$$\begin{aligned} \textrm{div} (\varrho \varvec{u}) = \nabla \varrho \cdot \varvec{u} + \varrho \textrm{div} \varvec{u} = \frac{\varrho }{{c_M}} \begin{pmatrix} x \\ y \end{pmatrix} \cdot \begin{pmatrix} -y \\ x \end{pmatrix} + 0 = 0. \end{aligned}$$

Hence, $(\varvec{u}, \varrho )$ is a solution of (25).

**Fig. 13:**
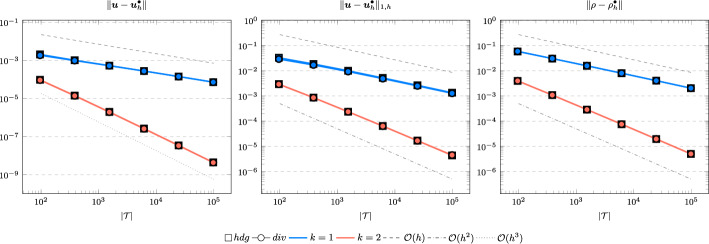
Convergence rates for $k \in \lbrace 1,2 \rbrace$ for the Navier–Stokes example with $\nu = 1$ and $c_M = 1$

**Fig. 14:**
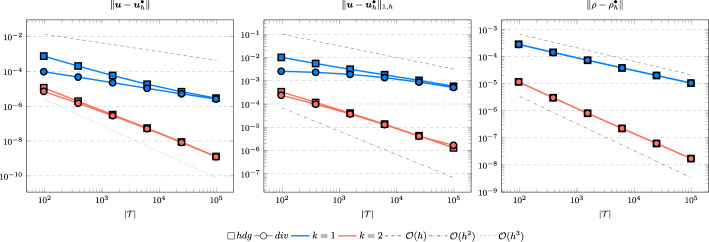
Convergence rates for $k \in \lbrace 1,2 \rbrace$ for the Navier–Stokes example with $\nu = 1$ and $c_M = 100$

**Fig. 15:**
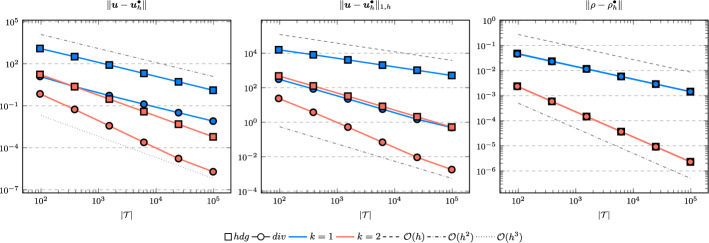
Convergence rates for $k \in \lbrace 1,2 \rbrace$ for the Navier–Stokes example with $\nu = 10^{-6}$ and $c_M = 1$

**Fig. 16:**
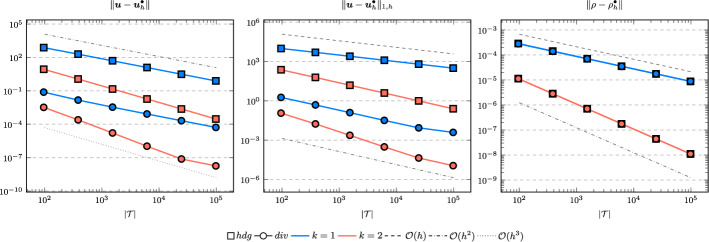
Convergence rates for $k \in \lbrace 1,2 \rbrace$ for the Navier–Stokes example with $\nu = 10^{-6}$ and $c_M = 100$

To mimic this situation, we solve the compressible Stokes problem with the right-hand side $\varvec{g}(x,y):= \nabla (x^2 + y^2) / 2$ and consider as before the cases $\nu \in \lbrace 1,10^{-6} \rbrace$, ${c_M}\in \lbrace 1,100 \rbrace$ and $k \in \lbrace 1,2 \rbrace$. Further note, that this example also requires non-homogeneous boundary data which we prescribe as Dirichlet boundary conditions for $\varvec{u}$ and, where $\varvec{u} \cdot \varvec{n}$ points into the domain, as a boundary inflow term for $\varrho$ in the continuity equation. We can make the following observations:

- For the case $\nu = 1$, see Fig. 13 for ${c_M}= 1$ and Fig. 14 for ${c_M}= 100$ an interesting observation can be made for the lowest order approximation. Although the discrete $H^1$-error and the density error converge with optimal orders, the $L^2$-error of the velocity only gives a linear convergence rate. In contrast to that we see, that the quadratic approximation converges optimally, i.e. with a cubic rate for the $L^2$-error of the velocity and a quadratic rate for all other errors.
- For the case of a vanishing viscosity $\nu = 10^{-6}$, see Fig. 15 for ${c_M}= 1$ and Fig. 16 for ${c_M}= 100$, we can make the same conclusions as for the previous examples. All errors converge with optimal order or show some pre-asymptotic faster convergence rate, and the gradient-robust solution $\varvec{u}_h^{div}$ provides a much better approximation.

## Data Availability

The datasets and scripts to generate the data with the finite element library NGSolve www.ngsolve.org are available from the corresponding author and at [29].

## References

[1] Akbas M, Gallouët T, Gassmann A, Linke A, Merdon C (2020). A gradient-robust well-balanced scheme for the compressible isothermal Stokes problem. Comput. Methods Appl. Mech. Eng..

[2] Audusse E, Bouchut F, Bristeau M-O, Klein R, Perthame B (2004). A fast and stable well-balanced scheme with hydrostatic reconstruction for shallow water flows. SIAM J. Sci. Comput..

[3] Berberich, J.P., Chandrashekar, P., Klingenberg, C.: High order well-balanced finite volume methods for multi-dimensional systems of hyperbolic balance laws (2020)

[4] Boffi D, Brezzi F, Fortin M (2013). Mixed Finite Element Methods and Applications.

[5] Brenner SC (2004). Korn’s inequalities for piecewise H1 vector fields. Math. Comput..

[6] Brenner SC (2003). Poincaré-Friedrichs inequalities for piecewise H1 functions. SIAM J. Numer. Anal..

[7] Cockburn B, Nguyen NC, Peraire J (2010). A comparison of HDG methods for Stokes flow. English. J. Sci. Comput..

[8] Cockburn B, Gopalakrishnan J, Lazarov R (2009). Unified hybridization of discontinuous Galerkin, mixed, and continuous Galerkin methods for second order elliptic problems. SIAM J. Numer. Anal..

[9] Cockburn B, Kanschat G, Schötzau D (2007). A note on discontinuous Galerkin divergence-free solutions of the Navier-Stokes equations. J. Sci. Comput..

[10] Cotter CJ, Thuburn J (2014). A finite element exterior calculus framework for the rotating shallow-water equations. J. Comput. Phys..

[11] Di Pietro DA, Ern A (2011). Mathematical aspects of discontinuous Galerkin methods.

[12] Falk RS, Neilan M (2013). Stokes complexes and the construction of stable finite elements with pointwise mass conservation. SIAM J. Numer. Anal..

[13] Feireisl, E., Lukáčová-Medviďová, M., Nečasová, V., Novotný, A., She, B.: Asymptotic preserving error estimates for numerical solutions of compressible Navier-Stokes equations in the low Mach number regime. Multiscale Model. Simul. **16**(1), 150–183 (2018)

[14] Gallouët T, Herbin R, Latché J-C (2009). A convergent finite element-finite volume scheme for the compressible Stokes problem. Part I: the isothermal case. Math. Comp..

[15] Gauger NR, Linke A, Schroeder PW (2019). On high-order pressure-robust space discretisations, their advantages for incompressible high Reynolds number generalised Beltrami flows and beyond. en. SMAI j. comput. math..

[16] Greenberg JM, Leroux AY (1996). A Well-Balanced Scheme for the Numerical Processing of Source Terms in Hyperbolic Equations. SIAM J. Numer. Anal..

[17] Grosheintz-Laval L, Käppeli R (2020). Well-balanced finite volume schemes for nearly steady adiabatic flows. J. Comput. Phys..

[18] Guzmán J, Neilan M (2014). Conforming and divergence-free Stokes elements on general triangular meshes. Math. Comp..

[19] Guzmán J, Shu C-W, Sequeira FA (2017). $${\rm H(div)}$$ conforming and DG methods for incompressible Euler’s equations. IMA J. Numer. Anal..

[20] John V, Li X, Merdon C, Rui H (2024). Inf-sup stabilized Scott-Vogelius pairs on general simplicial grids by Raviart-Thomas enrichment. Math. Models Methods Appl. Sci..

[21] John V, Linke A, Merdon C, Neilan M, Rebholz LG (2017). On the divergence constraint in mixed finite element methods for incompressible flows. SIAM Rev..

[22] Kikuchi F (2012). Rellich-type discrete compactness for some discontinuous Galerkin FEM. Jpn. J. Ind. Appl. Math..

[23] Kogler L, Lederer PL, Schöberl J (2023). A conforming auxiliary space preconditioner for the mass conserving stress-yielding method. Numer. Linear Algebra Appl..

[24] Lederer P, Linke A, Merdon C, Schöberl J (2017). Divergence-free reconstruction operators for pressure-robust stokes discretizations with continuous pressure finite elements. SIAM J. Numer. Anal..

[25] Lederer PL, Lehrenfeld C, Schöberl J (2018). Hybrid discontinuous Galerkin methods with relaxed $$H({\rm div})$$-conformity for incompressible flows. Part I. SIAM J. Numer. Anal..

[26] Lederer, P.L., Lehrenfeld, C., Stocker, P.: Trefftz discontinuous Galerkin discretization for the Stokes problem. In: Numerische Mathematik (10th Apr. 2024)

[27] Lederer PL, Schöberl J (2018). Polynomial robust stability analysis for H(div)-conforming finite elements for the Stokes equations. IMA J. Numer. Anal..

[28] Lederer, P.L., Stenberg, R.: Analysis of weakly symmetric mixed finite elements for elasticity. In: (to appear) Math. Comp. (2023)

[29] Lederer, P., Merdon, C.: Computational results for the work ”Gradient- robust hybrid DG discretizations for the compressible Stokes equations”. (Nov. 2023) 10.1007/s10915-024-02605-2 PMC11224105 38974937

[30] Lehrenfeld, C.: Hybrid Discontinuous Galerkin Methods for Incompressible Flow Problems. MA thesis. RWTH Aachen (2010)

[31] Linke A, Merdon C (2016). Pressure-robustness and discrete Helmholtz projectors in mixed finite element methods for the incompressible Navier-Stokes equations. Comput. Methods Appl. Mech. Engrg..

[32] Linke A (2012). A divergence-free velocity reconstruction for incompressible flows. C. R. Math. Acad. Sci. Paris.

[33] Linke A, Matthies G, Tobiska L (2016). Robust arbitrary order mixed finite element methods for the incompressible stokes equations with pressure independent velocity errors. ESAIM: M2AN.

[34] Mao S, Xue W (2023). Convergence and error estimates of a mixed discontinuous Galerkin-finite element method for the semi-stationary compressible stokes system. J. Sci. Comput..

[35] Michel-Dansac V, Berthon C, Clain S, Foucher F (2016). A well-balanced scheme for the shallow-water equations with topography. Comput. Math. Appl..

[36] Berberich JP, Käppeli R, Chandrashekar P, Klingenberg C (2021). High order discretely well-balanced methods for arbitrary hydrostatic atmospheres. Commun. Comput. Phys..

[37] Rhebergen S, Wells GN (2018). A hybridizable discontinuous Galerkin method for the Navier-Stokes equations with pointwise divergence-free velocity field. J. Sci. Comput..

[38] Schroeder PW, Lube G (2018). Divergence-free $$H({\rm div})$$-FEM for time-dependent incompressible flows with applications to high Reynolds number vortex dynamics. J. Sci. Comput..

[39] Scott, L.R., Vogelius, M.: Conforming finite element methods for incompressible and nearly incompressible continua. In: Large-scale computations in fluid mechanics, Part 2 (La Jolla, Calif., 1983). Vol. 22. Lectures in Appl. Math. Providence, RI: Amer. Math. Soc., pp. 221–244 (1985)

